# Propagating synchrony in feed-forward networks

**DOI:** 10.3389/fncom.2013.00153

**Published:** 2013-11-15

**Authors:** Sven Jahnke, Raoul-Martin Memmesheimer, Marc Timme

**Affiliations:** ^1^Network Dynamics, Max Planck Institute for Dynamics and Self-Organization (MPIDS)Göttingen, Germany; ^2^Bernstein Center for Computational Neuroscience (BCCN)Göttingen, Germany; ^3^Fakultät für Physik, Georg-August-Universität GöttingenGöttingen, Germany; ^4^Department for Neuroinformatics, Donders Institute, Radboud UniversityNijmegen, Netherlands

**Keywords:** synchrony, networks, synfire chains, spike pattern, mathematical neuroscience, non-additive coupling, non-linear dendrites

## Abstract

Coordinated patterns of precisely timed action potentials (spikes) emerge in a variety of neural circuits but their dynamical origin is still not well understood. One hypothesis states that synchronous activity propagating through feed-forward chains of groups of neurons (synfire chains) may dynamically generate such spike patterns. Additionally, synfire chains offer the possibility to enable reliable signal transmission. So far, mostly densely connected chains, often with all-to-all connectivity between groups, have been theoretically and computationally studied. Yet, such prominent feed-forward structures have not been observed experimentally. Here we analytically and numerically investigate under which conditions diluted feed-forward chains may exhibit synchrony propagation. In addition to conventional linear input summation, we study the impact of non-linear, non-additive summation accounting for the effect of fast dendritic spikes. The non-linearities promote synchronous inputs to generate precisely timed spikes. We identify how non-additive coupling relaxes the conditions on connectivity such that it enables synchrony propagation at connectivities substantially lower than required for linearly coupled chains. Although the analytical treatment is based on a simple leaky integrate-and-fire neuron model, we show how to generalize our methods to biologically more detailed neuron models and verify our results by numerical simulations with, e.g., Hodgkin Huxley type neurons.

## 1. Spike patterns and signal transmission in neuronal circuits

Reliable signal transmission is a core part of neuronal processing. A common hypothesis states that activity propagating along neuronal sub-populations that are connected in a feed-forward manner may support such signal transmission. Indeed, there is strong indication that activity propagation along feed-forward structures drives the generation of bird songs (Long et al., 2010) and experiments have shown propagation of synchronous and rate activity in feed-forward networks (FFNs) *in vitro* (Reyes, 2003; Feinerman et al., 2005; Feinerman and Moses, 2006). Sequential replay in the hippocampus and in neocortical networks also suggest underlying feed-forward mechanisms (August and Levy, 1999; Nadasdy et al., 1999; Lee and Wilson, 2002; Leibold and Kempter, 2006; Xu et al., 2011; Eagleman and Dragoi, 2012; Jahnke et al., 2012) and propagation of synchronous activity along feed-forward chains is a possible explanation for experimentally observed precise spike timing in the cortex (Riehle et al., 1997; Kilavik et al., 2009; Putrino et al., 2010). Further, the modular, hierarchical structure of many sensory and motor systems suggests propagation over sequences of areas in feed-forward manner, e.g., in bottom-up signal transfer (Felleman and Van Essen, 1991; Scannell et al., 1999; Bullmore and Sporns, 2009; Kumar et al., 2010).

Feed-forward structures which support the propagation of synchronous activity are termed synfire chains. The concept was introduced by Abeles (1982) as groups of neurons (layers) with dense anatomical connections between subsequent groups that are embedded in otherwise roughly randomly connected local neural circuits. Two major questions regarding the dynamical options for synfire activity include a) how synchrony may actively propagate and b) how such spatio-temporally coordinated spike timing may be robust against irregular background activity, because the synfire chains are part of a cortical network with dynamics defined by the so-called irregular balanced state (van Vreeswijk and Sompolinsky, 1996, 1998).

Addressing these points, theoretical studies have established conditions for stable propagation of synchrony in synfire chains (Diesmann et al., 1999; Gewaltig et al., 2001). Most synfire chain models assume functionally relevant FFNs that exhibit a very dense, often all-to-all connectivity between subsequent layers (Aviel et al., 2003; Mehring et al., 2003; Kumar et al., 2008) (see also a recent review on this topic Kumar et al., 2010). Such highly prominent feed-forward-structures, however, have not been found experimentally. Since cortical neural networks are overall sparse (e.g., Braitenberg and Schüz, 1998; Holmgren et al., 2003), we may also expect some level of dilution for embedded feed-forward chains. So far, computational model studies assumed that such chains created from existing connections in sparse recurrent networks exhibit strong synaptic efficiencies and specifically modified neuron properties to enable synchrony propagation (Vogels and Abbott, 2005).

Recently, we have shown that non-additive dendritic interactions promote propagation of synchrony (Jahnke et al., 2012). The non-additive dendritic interactions considered are mediated by fast dendritic spikes (Ariav et al., 2003; Gasparini et al., 2004; Polsky et al., 2004; Gasparini and Magee, 2006): upon stimulation within a time interval less than a few milliseconds, dendrites are capable of generating sodium spikes. These induce a strong, short and stereotypical depolarization in the soma. If this depolarization elicits a somatic spike, the spike occurs a fixed time interval after stimulation with sub-millisecond precision. This dendritic non-linearities relax the requirement of dense feed-forward anatomy and thereby allow for robust propagation of synchrony even in *diluted* FFNs with synapses of moderate strength within the biologically observed range.

In the present article, we analytically and numerically investigate in detail under which conditions synchronous activity may reliably propagate along the layers of an FFN where the inter-group connectivity is diluted, as may be expected when they are part of a sparse cortical network. An embedding network is mimicked by external, noisy input. We study the influence of the network setup, including the influence of the emulated embedding network, and of different types of standard linearly additive as well as non-additive dendritic interactions.

We derive analytical estimates for the critical connectivity—the minimal connectivity that allows robust propagation of synchrony. Some fundamental analytical results, in particular the ansatz for deriving a critical connectivity in the first place, have been briefly reported before (Jahnke et al., 2012). Here, we extend the approach and show how the bifurcation point, i.e., the transition point from the non-propagating to the propagating regime, can be estimated quantitatively from the neurons' ground state properties. We investigate the validity range of the analytical predictions and check them via direct numerical simulations. Furthermore, we discuss the applicability of our results to biologically more detailed neuron models and network setups. In particular, we argue that the assumptions underlying the analytical approach are met by a wide class of neuron models, including, e.g., conductance based leaky integrate-and-fire and Hodgkin–Huxley-type neurons.

The article is structured as follows: After introducing the neuron model and network setup in section 2, we study in the main part the propagation of synchrony in linearly coupled FFNs (section 3.1) and in FFNs incorporating dendritic non-linearities (section 3.2). In particular, we derive tools to study the system analytically, compare the results to computer simulations and elaborate differences of the dynamics of FFNs with and without non-additive dendritic interactions. In the final part (section 3.3), we discuss the application of our analytical results to biologically more detailed neuron models.

## 2. Methods and models

### 2.1. Neuron model

#### 2.1.1. Linear model

Consider networks of leaky integrate-and-fire neurons that interact by sending and receiving spikes via directed connections. The state of neuron *k* at time *t* is described by its membrane potential *V*_*k*_(*t*) and its dynamics satisfy

(1) $$\frac{dV_{k}(t)}{dt}=-\frac{V_{k}(t)}{\tau_{k}^{\text{m}}}+I_{k}^{\text{const}}+I_{k}^{\text{net}}(t)+I_{k}^{\text{ext}}(t),$$

where τ^m^_*k*_ is the membrane time constant of neuron *k, I*^const^_*k*_ := *I*^0^_*k*_/τ^m^_*k*_ a constant input current, *I*^net^_*k*_(*t*) the input current caused by spikes within the network and *I*^ext^_*k*_(*t*) the input current arising from spikes from external sources. When the neuron's membrane potential reaches or exceeds the threshold Θ_*k*_ its membrane potential is reset to *V*^reset^_*k*_ and a spike is sent to the postsynaptic neurons *n*, where it changes the postsynaptic potential after a delay τ_*nk*_. After emitting a spike at *t* = *t*_0_ the neuron becomes refractory for a time period *t*^ref^, i.e., *V*_*k*_(*t*) = *V*^reset^_*k*_ for *t* ϵ [*t*_0_, *t*_0_ + *t*^ref^].

To keep the model analytically tractable, we model the fast rise of the membrane potential upon the arrival of presynaptic spikes by instantaneous jumps of the membrane potential, such that the resulting input current reads

(2) $$I_{k}^{\text{net}}(t)=\sum_{l}\sum_{m}\epsilon_{kl}\delta \left(t-t_{lm}^{f}-\tau_{kl}\right).$$

Here ϵ_*kl*_ denotes the coupling strength from neuron *l* to neuron *k, t*^*f*^_*lm*_ is the *m*th spike time of neuron *l* and τ_*kl*_ specifies the synaptic delay. In addition to spikes from the network each neuron receives excitatory and inhibitory random inputs that emulate an embedding network. These external inputs are modeled as random Poisson spike trains with rate ν^exc^ and ν^inh^, respectively. The resulting input current is given by

(3) $$I_{k}^{\text{ext}}(t)=\sum_{m}\epsilon^{\text{exc}}\delta \left(t-t_{km}^{\text{ext},\text{exc}}\right)+\sum_{m}\epsilon^{\text{inh}}\delta \left(t-t_{km}^{\text{ext},\text{inh}}\right),$$

where *t*^ext, exc^_*km*_ (*t*^ext, inh^_*km*_) is the arrival time of the *m*th excitatory (inhibitory) spike to neuron *k* and ϵ^exc^ > 0 (ϵ^inh^ < 0) denote the corresponding coupling strength.

#### 2.1.2. Non-linear model

In the above model all input currents are summed up linearly. To also investigate the effect of dendritic spikes we modulate the sum of synchronously arriving excitatory inputs by a non-linear dendritic modulation function σ_*NL*_ (·). This can be directly read off from experimental data (Ariav et al., 2003; Gasparini et al., 2004; Polsky et al., 2004; Gasparini and Magee, 2006): If the sum of excitatory inputs is below the dendritic threshold Θ_*b*_, the single inputs are processed linearly (σ_*NL*_ (·) equals the identity). If the sum of inputs exceeds the dendritic threshold Θ_*b*_, the depolarization is strongly non-linearly enhanced compared to that expected from linear summation. This is, in biological terms, due to a dendritic spike elicited. Larger inputs have been experimentally found to not further increase the somatic peak depolarization. The dendritic modulation function may then be modeled as

(4) $$\sigma_{NL}\left(\epsilon \right)=\{\begin{array}{ll} \epsilon & \text{for }\epsilon <\Theta_{b}\\ \kappa & \text{otherwise} \end{array}.$$

The dendrites process synchronous inputs non-additively: inputs below the dendritic threshold are summed linear, inputs above this threshold are summed supra-linear and, due to the saturation, very large inputs are summed sub-linear.

If not stated otherwise, we consider only exactly simultaneous arriving spikes as sufficiently synchronous; to allow for exactly simultaneous arrivals, the synaptic delays are chosen as homogeneous τ_*kl*_ ≡ τ. The input currents caused by spikes that are received from the network are then given by

(5) $$I_{k}^{\text{net}}(t)\;=\sum_{t^{f}}\left[\sigma_{NL}\left(\sum_{l\;\in M_{\text{exc}}\left(t^{f}\right)}\epsilon_{kl}\right)+\sum_{l\in M_{\text{inh}}\left(t^{f}\right)}\epsilon_{kl}\right]\delta \left(t-t^{f}-\tau \right).\;\;\;\;\;$$

Here, the sum over *t*^*f*^ denotes the sum over all times at which spike(s) are sent in the network, irrespective of which neuron(s) is (are) spiking. The sets *M*_exc_(*t*^*f*^) and *M*_inh_(*t*^*f*^) specify the set of neurons that send an excitatory or inhibitory spike at time *t*^*f*^, respectively. (To describe a network with linear dendrites σ_*NL*_(ϵ) is replaced by ϵ).

In section 3.3.1 we consider inhomogeneous delay distributions and finite dendritic integration window Δ*t* (i.e., non-linear amplification of inputs received within finite time interval Δ*t*) and discuss how the results achieved for homogeneous systems can be generalized.

### 2.2. Network topology

We consider the propagation of synchrony in diluted Feed-Forward-Networks (FFNs, synfire-chains). They consist of a sequence of *m* layers, each composed of ω neurons. Neurons of one layer form excitatory projections to the neurons of the subsequent layer with probability *p*; the strength of an existing connection from neuron *l* to neuron *k* is denoted by ϵ_*kl*_.

For simplicity of presentation, we consider homogeneous neuronal populations, i.e., all neurons have identical properties (τ^m^_*k*_ = τ^m^, Θ_*k*_ = Θ and *V*^reset^_*k*_ = *V*^reset^ for all *i*), as well as homogeneous coupling strengths, i.e., ϵ_*kl*_ = ϵ if a connection is realized, throughout this article. If not stated otherwise, we use τ^m^ = 14 ms and Θ = 15 mV as standard values for the membrane time constant and the neuron threshold.

### 2.3. Ground state dynamics

We consider networks, where the single neurons are placed in a “fluctuation driven regime,” i.e., in the ground state the average input to each neuron is sub-threshold and spiking of neurons is caused by fluctuations of the inputs. This setup allows to emulate the dynamics of neurons which are part of a balanced network (van Vreeswijk and Sompolinsky, 1996, 1998). The neurons fire asynchronously and irregularly with low firing rate ν; the spike trains resemble Poissonian spike trains (Tuckwell, 1988; Brunel and Hakim, 1999; Brunel, 2000; Burkitt, 2006). Thus, the inputs to the neurons may be described by three Poissonian spike trains with rates ν^exc^ (external, excitatory), ν^inh^ (external, inhibitory) and ν^int^ = ν*p*ω (inputs from the preceding layer). Since the number of inputs *N*^*X*^_*T*_, *X* ∈ {exc, inh, int}, in a time interval *T* is Poisson distributed, the expected number of inputs 〈*N*^*X*^_*T*_〉 and the variance 〈 (*N*^*X*^_*T*_ − 〈*N*^*X*^_*T*_〉)^2^〉, equal ν^*X*^*T* = 〈*N*^*X*^_*T*_〉 = 〈(*N*^*X*^_*T*_ − 〈*N*^*X*^_*T*_〉)^2^〉.

Then

(6) $$\mu =I_{0}+\tau^{\text{m}}\nu^{\text{exc}}\epsilon^{\text{exc}}+\tau^{\text{m}}\nu^{\text{inh}}\epsilon^{\text{inh}}+\tau^{\text{m}}p\omega \nu \epsilon$$

is the mean of the total input to the neurons in an interval of the size of the membrane time constant, *T* = τ^m^, and

(7) $$\sigma^{2}=\tau^{\text{m}}\nu^{\text{exc}}{\left(\epsilon^{\text{exc}}\right)}^{2}+\tau^{\text{m}}\nu^{\text{inh}}{\left(\epsilon^{\text{inh}}\right)}^{2}+\tau^{\text{m}}p\omega \nu \epsilon^{2}$$

is its variance. In diffusion approximation, the distribution of membrane potentials *P*_*V*_(*V*) and the mean firing rate ν can be derived analytically (Brunel and Hakim, 1999; Brunel, 2000; Helias et al., 2010). In particular, for networks with low firing rates the probability density of membrane potentials (see, e.g., Tuckwell, 1988)

(8) $$P_{V}(V)=\frac{1}{\sqrt{\pi \sigma^{2}}}\exp\text{​}\left[-{\left(\frac{V-\mu }{\sigma }\right)}^{2}\right]$$

is Gaussian and can be expressed in terms of the input current. In this approximation the average firing rate is

(9) $$\nu =\frac{1}{\sqrt{\pi }\tau^{\text{m}}}\frac{\Theta -\mu }{\sigma }\exp\text{​}\left[-{\left(\frac{\Theta -\mu }{\sigma }\right)}^{2}\right]$$

and depends on μ and σ only via the quotient

(10) $$\alpha :=\frac{\Theta -\mu }{\sigma },$$

which is the distance of the average input μ from the neurons' threshold Θ normalized by the standard deviation σ of the input. For the analytical derivations throughout this article we focus on the regime of low spiking rates (α ⪆ 2; ν ⪅ 1.5Hz).

In the absence of synchronous activity each neuron receives a large number of inputs from the external network and only a few inputs from the previous layer of the FFN, such that the ground state dynamics of the network is mainly established by the external inputs. To keep the input balanced we choose ν^exc^ = ν^inh^ = :ν^ext^ and ϵ^exc^ = −ϵ^inh^ = :ϵ^ext^ throughout the article.

### 2.4. Propagation of synchrony

**Figure 1:**
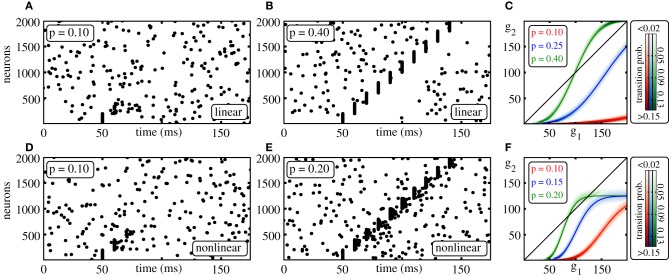
**Propagation of synchrony in diluted FFNs. (A,B,D,E)** Raster plots of diluted feed-forward networks (*m* = 10, ω = 200, ϵ = 0.25 mV). With increasing connection probability *p* propagation of synchrony can be enabled **(A,B)** in networks with additive (linear) and **(D,E)** in networks with non-additive (non-linear) dendritic interactions (Θ_*b*_ = 4 mV, κ = 11 mV). **(C,F)** Average number of synchronously active neurons in the second layer, *g*_2_, vs. the number of synchronously active neurons in the initial layer, *g*_1_; panel **(C)** linear, panel **(F)** non-additive dendritic interactions (average over *n* = 10,000 trials: solid line, transition probability: shading). Note that non-linear dendrites allow for sparser connectivity, **(E)** vs. **(B)** and for a sparser code, i.e., for smaller numbers of spiking neurons in an activated group, **(F)** vs. **(C)**.

To initiate propagating synchronous activity along the considered diluted FFN, we excite in the first layer a subgroup of *g*_0_ ≤ ω neurons to spike synchronously. This causes a synchronous input to the following layer after the synaptic delay τ and may therefore initiate synchronous spiking of a subgroup of neurons in that layer. These may again excite synchronous spiking in the next layer and so on. Depending on the ground state, i.e., the layout of the external network, on the layer size ω, and on the coupling strength ϵ, a synchronous pulse may or may not propagate along the FFN (cf. Figures 1A,B,D,E).

In addition to the triggered propagation, one might generally also expect the occurrence of spontaneous propagation of synchronous activity: Neurons of a particular layer share inputs from the previous layer and this causes correlations in their spiking activity. Over the layers these correlations can accumulate and lead to synchronous spiking (Aviel et al., 2003; Rosenbaum et al., 2010, 2011; Litvak et al., 2013). However, in the setups considered in this article, the effect is negligible due to two reasons: (1) each neuron receives a large number of external (uncorrelated) inputs and this background noise has a decorrelating effect, (2) we study the system near the critical point, i.e., for parameters where even synchronized spiking of all neurons of a particular layer is just sufficient to initiate a propagation of synchrony. Thus, spontaneous propagation of synchrony effectively does not occur.

We study the transition from the non-propagating to the propagating regime by means of a iterated map that yields the expectation value of the number of synchronously spiking neurons *g*_*i* + 1_ in layer *i* + 1 if *g*_*i*_ neurons are synchronously active in layer *i*. There is always one trivial fixed point, *G*_0_, of this iterated map with 0 = *G*_0_ = *g*_*i* + 1_ = *g*_*i*_, which corresponds to absent activity. If *g*_*i* + 1_ < *g*_*i*_ for all *g*_*i*_ > *G*_0_, synchronous activity will die out after a small number of layers. If *g*_*i* + 1_ ≥ *g*_*i*_ for some substantial group size, *g*_*i*_ > *G*_0_, a stable propagation of synchrony may be enabled (cf. Figures 1C,F). More precisely, we will show in this article that with increasing connectivity *p* the system undergoes a tangent bifurcation and two fixed points *G*_1_ and *G*_2_ ≥ *G*_1_ appear. If existing, *G*_1_ is always unstable (the diagonal is crossed from below; the slope of the iterated map needs to be larger than one) and *G*_2_ is always stable [all connections within the FFN are excitatory such that the iterated map is monotonically increasing (slope larger than zero, in particular larger than −1)]; further at *G*_2_ there is an intersection with the diagonal from above thus the slope is smaller than one and stationary propagation with group sizes around *G*_2_ is enabled.

In computer simulations, we determine for each given network setup by the following procedure whether a propagation is possible: after some initial time *t*^init^ we excite all neurons of the first layer to spike synchronously and measure the number of active neurons *g*_*i*_ in the *i*th layer at the expected spiking time *t*^exp^_*i*_ = *t*^init^ + *i*τ. If *g*_*i*_ is substantially larger than the number of active neurons arising from spontaneous activity in more than 50% of *n* trials (i.e., *n* repetition of the same simulation with different initial conditions), we denote the propagation of synchrony as successful. The critical connectivity p^*^, that marks the transition from a regime where propagation of synchrony is not possible to a regime where propagation of synchrony is enabled, is found by determining the lowest connection probability *p* for which an initial synchronous pulse propagates successfully.

As the connections within the FFN are all excitatory, it is sufficient to check whether propagation of synchrony can be initiated by inducing synchronized spiking of all ω neurons of the first layer: Stationary propagation of synchrony can be enabled if there is a non-trivial stable fixed point (*G*_2_) of the iterated map for the average group size. For purely excitatory connections the basin of attraction of this fixed point is bounded from the left by an unstable fixed point (*G*_1_) and from the right by the maximum group size given by the layer size ω.

## 3. Results and discussion

Under which conditions can synchronous signals propagate robustly along diluted FFNs? To answer this question in detail, we first focus on networks with linear dendrites. Afterwards we study the propagation of synchrony in networks incorporating non-additive dendritic interactions and compare with the linear case. Finally, we show that the derived results are directly applicable in biologically more detailed neuron models and network configurations.

### 3.1. FFNs with linear dendrites

In this section, we consider linearly coupled FFNs. In the first part, we derive analytical estimates for the critical connectivity p^*^_L_ that marks the transition from the non-propagating to the propagating regime; the initial steps follow the lines of Jahnke et al. (2012); Memmesheimer and Timme (2012). In the second part we investigate the influence of the external network on the propagation of synchrony and determine the parameter-region for which the analytical estimates are applicable. In particular, we show that the derived estimates are applicable in the biologically relevant parameter-region, where the spontaneous firing rate is low and the distribution of membrane potentials is sufficiently broad. Finally, we study how the properties of propagating synchronous pulses depend on different system parameters.

#### 3.1.1. Analytical derivation of critical connectivity

To access the properties of propagation of synchrony we consider average numbers of active neurons in the different layers of an FFN: for this, we derive a iterated map which yields the expected number of neurons that will spike synchronously in one layer given that in the preceding layer a certain number of neurons was synchronously active.

If in the *i*th layer, *g*_*i*_ neurons spike synchronously, the number of synchronous inputs *h* a single neuron in layer *i* + 1 receives follows a binomial distribution *h* ~ *B*(*g*_*i*_, *p*). We denote the spiking probability of a single neuron due to an input of strength *x* by *p*_*f*_(*x*). The average or expected spiking probability *p*^sp^(*g*_*i*_) of a single neuron in layer *i* + 1 is then given by

(11) $$p^{\text{sp}}\left(g_{i}\right)=\text{E}\left[p_{f}\left(h\epsilon \right)|g_{i}\right]=\sum_{h=0}^{g_{i}}\left(\begin{array}{c} g_{i}\\ h \end{array}\right)p^{h}{\left(1-p\right)}^{g_{i}-h}p_{f}\left(h\epsilon \right).$$

Here and in the following we denote the expectation value of a function *f*(*X*) of a random variable *X* by E [*f*(*X*)]; conditional expectations are denoted by E [*f*(*X*)|*Y*]. The expected number of spiking neurons in layer *i* + 1 is then simply

(12) $$\text{E}\left[g_{i+1}|g_{i}\right]=\omega p^{\text{sp}}\left(g_{i}\right)$$

(13) $$=\omega \sum_{h=0}^{g_{i}}\left(\begin{array}{c} g_{i}\\ h \end{array}\right)p^{h}{\left(1-p\right)}^{g_{i}-h}p_{f}\left(h\epsilon \right).$$

If the connection probability *p* is low and/or the connection strengths ϵ are small, the spontaneous spiking activity in the absence of synchrony is only weakly influenced by the spiking activity within the FFN. Thus as a starting point, we assume that the ground state is exclusively governed by external inputs (effectively setting ϵ_*ij*_ ≡ 0). Then, the mean input to the neurons in an interval of length τ^m^ is μ = *I*_0_ with standard deviation $\sigma =\epsilon^{\text{ext}}\sqrt{2\tau^{\text{m}}\nu^{\text{ext}}}$ (cf. section 2.3). Using the probability density (Equation 8), we calculate the spiking probability of a single neuron, *p*_*f*_(*x*), due to an input of strength *x*;

(14) $$p_{f}\left(x\right)=\int_{\Theta -x}^{\Theta }P_{V}\left(V\right)dV$$

(15) $$=\frac{1}{2}\left(\text{Erf}\left[\frac{\Theta -\mu }{\sigma }\right]-\text{Erf}\left[\frac{\Theta -\mu +x}{\sigma }\right]\right)$$

equals the probability of finding a neuron's membrane potential in the interval [Θ − *x*, Θ]. To derive a iterated map for the average number of active neurons (which maps *E*[*g*_*i*_] → *E*[*g*_*i* + 1_]), we interpolate E [*g*_*i* + 1_ | *g*_*i*_] for continuous *g*_*i*_ and in the second step replace *g*_*i*_ by its expectation value E [*g*_*i*_]. The fixed points, E [*g*_*i* + 1_ | E [*g*_*i*_]] = E [*g*_*i*_], qualitatively determine the propagation properties of synchronous activity. In the rest of the manuscript we are dealing with the average number of active neurons in a given layer. Therefore, for simplicity we denote the expectation value of the average number of active neurons in a given layer *i* by *g*_*i*_ instead of E [*g*_*i*_].

For sufficiently small connection probabilities *p* the map (Equation 12) has only one (trivial) fixed point *G*_0_ = *g*_*i* + 1_ = *g*_*i*_ = 0. Any initial synchronous pulse will die out after a small number of layers (see also Figure 1). With increasing connectivity two additional fixed points *G*_1_ (unstable) and *G*_2_ ≥ *G*_1_ (stable) appear via a tangent bifurcation.

For FFNs with purely excitatory couplings between the layers, the second fixed point *G*_2_ (if it exists) is always stable: The spiking probability *p*_*f*_(*x*) is monotonically increasing with input *x* and thus also the iterated map (Equation 13) is monotonically increasing (i.e., the slope is larger than 0). Moreover, if *G*_2_ exists the slope of the iterated map at this intersection point with the diagonal is smaller than 1. This implies that *G*_2_ is stable and synchronous pulses of size *g*_*i*_ ≥ *G*_1_ typically initiate a propagation of synchrony with an average number of active neurons around *G*_2_. The critical connectivity p^*^_L_ at the bifurcation point marks the minimal connectivity that allows for stable propagation of synchrony.

Although the distribution of inputs from one layer to the subsequent one and the spiking probability of a single neuron *p*_*f*_(·) are known, there is no analytic closed form solution to the fixed point equation *g*_*i* + 1_ = *g*_*i*_ = g^*^_i_. In other words, we can compute the firing probability *p*_*f*_(*x*_0_) for any *x*_0_, and therefore also E [*g*_*i* + 1_ | *g*_*i*_] for any *g*_*i*_, but g^*^_i_ = E [*g*_*i* + 1_ | g^*^_i_] is transcendental. We thus derive an approximate solution. We choose some expansion point *g*_*i*_ (see section 3.1.2 for details), and approximate the function E [*g*_*i* + 1_ | g^*^_i_] by a polynomial *g*_*i*_ + *S*(g^*^_i_ − *g*_*i*_) in second order in (g^*^_i_ − *g*_*i*_) near *g*_*i*_. The arising quadratic fixed point equation g^*^_i_ = *g*_*i*_ + *S*(g^*^_i_ − *g*_*i*_) is then analytically solvable in g^*^_i_. This also allows to analytically compute the critical connectivity p^*^_L_: it is the parameter value at which the iterated map undergoes a tangent bifurcation, i.e., at which the two solutions of the fixed point equation become equal upon changing from complex-conjugate to real. Since the right hand side of Equation (13) does not offer itself for a direct series expansion in g^*^_i_, we derive *g*_*i*_ + *S*(g^*^_i_ − *g*_*i*_) from an appropriate expansion of *p*_*f*_(*h*ϵ) and a subsequent computation the arising expectation values.

In biologically relevant scenarios, the neurons usually receive a large number of synaptic inputs and thus the distribution of membrane potentials *P*_*V*_(*V*) is broad, *P*_*V*_(*V*) changes slowly with *V*. Then, *P*_*V*_(*V*) around some *V* = *V*_0_ can be approximated by considering a series expansion with a small order and it is possible to derive an approximation for the critical connectivity p^*^_L_ based on an expansion of *p*_*f*_(·). Expanding *p*_*f*_(*x*) into a Taylor series around some *x*_0_ and using Equation (12) yields

(16) $$g_{i+1}=\omega \text{E}\left[\sum_{n=0}^{\infty }\frac{p_{f}^{\left(n\right)}\left(x_{0}\right)}{n!}{\left(h\epsilon -x_{0}\right)}^{n}|g_{i}\right]$$

(17) $$=\omega \sum_{n=0}^{\infty }\frac{p_{f}^{\left(n\right)}\left(x_{0}\right)}{n!}\text{E}\left[{\left(h\epsilon -x_{0}\right)}^{n}|g_{i}\right].$$

Here and in the following we denote the *n*th derivative of a function *f*(*x*) at *x* = *x*_0_ by

(18) $$f^{\left(n\right)}\left(x_{0}\right)={\frac{d}{d^{n}x}f(x)|}_{x=x_{0}}.$$

Replacing the derivatives of *p*_*f*_(·) by the (one order lower) derivatives of probability density of membrane potentials *P*_*V*_(*V*) according to Equation (14) yields

(19) $$g_{i+1}=\omega p_{f}\left(x_{0}\right)+\omega \sum_{n=1}^{\infty }\frac{P_{V}^{\left(n-1\right)}\left(V_{0}\right)}{{\left(-1\right)}^{n-1}n!}\text{E}\left[{\left(h\epsilon -x_{0}\right)}^{n}|g_{i}\right],$$

where we defined

(20) $$V_{0}:=\Theta -x_{0}$$

for better readability.

We have recently shown (Jahnke et al., 2012) that it is possible to derive a scaling law for the critical connectivity using

(21) $$x_{0}=g_{i}p\epsilon ,$$

the (unknown) average input from one layer to the next during stationary synchrony propagation, as expansion point. For this choice the expectation value E [(*h*ϵ − *x*_0_)^*n*^ | *g*_*i*_] in Equation (19) simplifies to

(22) $$\text{E}\left[{\left(h\epsilon -x_{0}\right)}^{n}|g_{i}\right]=\epsilon^{n}\text{E}\left[{\left(h-\text{E}\left[h\right]\right)}^{n}|g_{i}\right]=\epsilon^{n}m_{n},$$

where we denote by *m*_*n*_ the *n*th central moment of the Binomial distribution *B*(*g*_*i*_, *p*), specifying the distribution of inputs to the (*i* + 1)th layer. In the limit of large layer sizes ω and small coupling strengths ϵ keeping the maximal input ϵω to each layer constant (to preserve the network state), all summands for *n* ≥ 2 vanish, and Equation (19) simplifies to

(23) $$g_{i+1}=\omega p_{f}\left(g_{i}p\epsilon \right).$$

Using the implicit function theorem one can show that this implies the scaling law

(24) $$p_{L}^{*}=\frac{1}{\lambda \epsilon \omega }$$

where λ is a constant independent of ϵ and ω (Jahnke et al., 2012). We note that for the derivation of the scaling law (Equation 24) we did not use the actual functional form of the distribution of membrane potentials *P*_*V*_(*V*). Therefore this estimate holds if *P*_*V*_(*V*) is sufficiently slow changing with *V* such that the Taylor expansion (cf. Equation 16) is applicable, but its validity is not restricted to the low-rate approximation.

However, the dependence of the prefactor 1/λ on the layout of the external network remained unknown. Here, we present an approach that enables us to derive an approximate value for λ. We consider the expansion (Equation 19) around *x*_0_ up to second order,

(25) $$\begin{array}{l} g_{i+1}\approx \omega p_{f}(x_{0})+\omega P_{V}\left(V_{0}\right)\cdot \left(\epsilon g_{i}p-x_{0}\right)\\ \;-\frac{\omega P_{V}^{\left(1\right)}(V_{0})}{2}\left[{\left(\epsilon g_{i}p-x_{0}\right)}^{2}+\epsilon^{2}g_{i}p\left(1-p\right)\right] \end{array}$$

The truncated series (Equation 25) is quadratic in *g*_*i*_ such that the fixed points g^*^_1/2_ = *g*_*i* + 1_ = *g*_*i*_ can be obtained analytically,

(26) $$g_{1,2}^{*}=\gamma_{L}\pm \sqrt{\gamma_{L}^{2}-\frac{x_{0}\left(2P_{V}(V_{0})+x_{0}P_{V}^{\left(1\right)}\left(V_{0}\right)\right)-2p_{f}(x_{0})}{p^{2}P_{V}^{\left(1\right)}(V_{0})\epsilon^{2}}},\;\;\;\;\;$$

where we defined

(27) $$\gamma_{L}:=\frac{p\epsilon \omega \left(2\left(P_{V}(V_{0})+x_{0}P_{V}^{\left(1\right)}(V_{0})\right)+\left(p-1\right)P_{V}^{\left(1\right)}(V_{0})\epsilon \right)-2}{2p^{2}P_{V}^{\left(1\right)}(V_{0})\epsilon^{2}\omega }.\;\;\;\;$$

At the bifurcation point, the root in Equation (26) vanishes such that both fixed points agree (g^*^_1_ = g^*^_2_) and γ_*L*_ = g^*^_1_ = g^*^_2_ specifies the average size of a propagating synchronous pulse. Consequently, the critical connectivity is obtained by choosing *p* such that

(28) $$\gamma_{L}^{2}=\frac{x_{0}\left(2P_{V}(V_{0})+x_{0}P_{V}^{\left(1\right)}\left(V_{0}\right)\right)-2p_{f}(x_{0})}{p^{2}P_{V}^{\left(1\right)}(V_{0})\epsilon^{2}}$$

which yields

(29) $$p_{L}^{*}=\frac{1}{2}-\frac{1}{\epsilon }\left[\frac{\lambda^{*}}{P_{V}^{\left(1\right)}(V_{0})}-\sqrt{\frac{2}{P_{V}^{\left(1\right)}(V_{0})\omega }+\frac{{\left(\epsilon P_{V}^{\left(1\right)}(V_{0})-2\lambda^{*}\right)}^{2}}{4{\left(P_{V}^{\left(1\right)}(V_{0})\right)}^{2}}}\right]$$

where we defined

(30) $$\begin{array}{l} \lambda^{*}:=P_{V}(V_{0})+x_{0}P_{V}^{\left(1\right)}(V_{0})\\ \;-\sqrt{P_{V}^{\left(1\right)}(V_{0})\left(x_{0}\left(2P_{V}(V_{0})+x_{0}P_{V}^{\left(1\right)}\left(V_{0}\right)\right)-2p_{f}(x_{0})\right)} \end{array}$$

which is independent of the setup of the FFN and completely determined by the layout of the external network and the choice of the expansion point *x*_0_.

As before we consider the limit of large layer sizes ω and small coupling strengths ϵ, i.e., we replace $\omega \to \frac{\text{const}}{\epsilon }$ and consider the leading terms of a series expansion of Equation (29). The expansion of the square bracket in Equation (29) yields

(31) $$\begin{array}{l} \frac{\lambda^{*}}{P_{V}^{\left(1\right)}(V_{0})}-\sqrt{\frac{2}{P_{V}^{\left(1\right)}(V_{0})}\frac{\epsilon }{\text{const}}+\frac{{\left(\epsilon P_{V}^{\left(1\right)}(V_{0})-2\lambda^{*}\right)}^{2}}{4{\left(P_{V}^{\left(1\right)}(V_{0})\right)}^{2}}}\\ =\left[\frac{\lambda^{*}}{P_{V}^{\left(1\right)}(V_{0})}-\frac{\lambda^{*}}{P_{V}^{\left(1\right)}(V_{0})}\right]-\epsilon \left(\frac{1}{\lambda^{*}\cdot \text{const}}-\frac{1}{2}\right)+O\left(\epsilon^{2}\right)\text{​}, \end{array}$$

such that the critical connectivity assumes the functional form given by Equation (24),

(32) $$p_{L}^{*}\approx \frac{1}{\lambda^{*}\epsilon \omega }.$$

Thus λ = λ^*^ defined by Equation (30) provides an approximation of the constant λ fully specifying the critical connectivity p^*^_L_.

#### 3.1.2. Optimal expansion point

**Figure 2:**
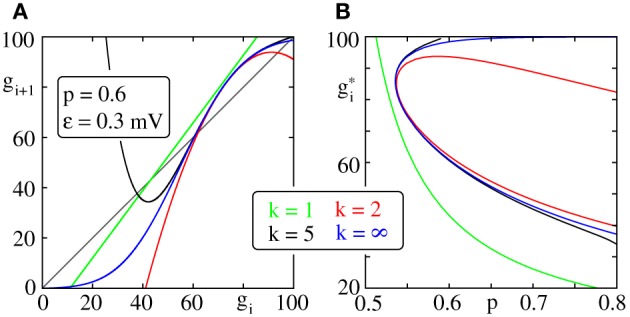
**Iterated map and bifurcation diagram for the average group size of a propagating synchronous pulse**. **(A)** Iterated map (Equation 19) truncated after expansion order *k* (color code) with *x*_0_ = x^*^_0_ (cf. Equation 37). **(B)** Fixed points of the iterated maps shown in **(A)**; with increasing connectivity two fixed points appear by a saddle node bifurcation. We note that already a second order expansion (red), i.e., the lowest order at which a saddle node bifurcation can occur, approximates the bifurcation diagram (blue) near the bifurcation point well.

To derive Equation (30) we assumed that it is sufficient to consider the second order expansion of *p*_*f*_(*x*). It is thus necessary to choose an appropriate expansion point that results in fast convergence. In particular for the choice *x*_0_ = x^*^_0_, that we will now derive, Equation (37) below, the bifurcation diagram near the bifurcation point is well approximated already for *k* = 2 (cf. Figure 2).

The size of a propagating group at the critical connectivity is γ_*L*_ (cf. Equation 27) and thus the resulting average input is p^*^_L_γ_*L*_ϵ. Our expansion point *x*_0_ should lie near to this value, which is, of course, unknown prior to solving the fixed point equation. We will thus compute a range in which p^*^_L_γ_*L*_ϵ has to lie and choose the expansion point appropriately within. We assume that ω is large and employ Equation (23) which allows an direct estimate of this range as we know the functional form explicitly. Equation (23) with *g*_*i* + 1_ = *g*_*i*_ is just another transcendental equation for the fixed points and it has zero, one, or two non-trivial fixed point solutions points g^*^_1_ and g^*^_2_, which are then also solutions of Equation (19) with *g*_*i* + 1_ = *g*_*i*_. At the bifurcation point (g^*^ = g^*^_1_ = g^*^_2_) where the diagonal is touched, the function *p*_*f*_(*gp*ϵ) has to be concave and monotonic increasing with respect to *g*. The definition (Equation 14) of *p*_*f*_(*x*) implies that it is monotonic increasing for all *x* ≥ 0. Moreover, it is concave for all *x* ≥ Θ − μ,

(33) $$p_{f}^{\left(1\right)}(x)=P_{V}(\Theta -x)\geq 0\text{ for }x\geq 0$$

(34) $$p_{f}^{\left(2\right)}(x)=-P_{V}^{\left(1\right)}(\Theta -x)\leq 0\text{ for }x\geq \Theta -\mu ,$$

such that the bifurcation point satisfies

(35) $$x_{0}\geq \Theta -\mu .$$

The condition Equation (33) holds because *P*_*V*_(*V*) ≥ 0 is a probability density and Equation (34) is derived directly from differentiating Equation (8). To maximize the quality of the second order approximation Equation (25), we choose *x*_0_ = x^*^_0_ such that the contribution to the expansion (Equation 19) of the *k* = 3rd order term equals zero. According to Equation (19), all 3rd order terms are proportional to *P*^(2)^_*V*_ (Θ − *x*_0_); so we determine the expansion point x^*^_0_ as a deflection point of *P*_*V*_(·), requiring that the second derivative of *P*_*V*_(*V*) vanishes for *V* = Θ − x^*^_0_,

(36) $${p_{f}^{\left(3\right)}(x_{0}^{*})=\frac{d^{2}P_{V}(V)}{dV^{2}}|}_{V=\Theta -x_{0}^{*}}\overset{!}{=}0.$$

In the considered regime of low spiking rates, we find $x_{0}^{*}=\Theta -\mu \pm \frac{\sigma }{\sqrt{2}}$, cf. Equation (8). Due to Equation (35)

(37) $$x_{0}^{*}=\Theta -\mu +\frac{\sigma }{\sqrt{2}}.$$

**Figure 3:**
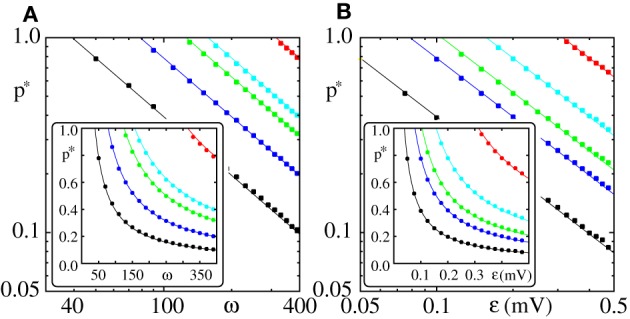
Critical connectivity p^*^_L_ in FFNs with linear dendrites decays algebraically with coupling strength ϵ and layer size ω. The parameters of the external inputs (emulated embedding network) are fixed (*I*_0_ = 5 mV, ν^ext^ = 3 kHz, ϵ^ext^ = 0.5 mV). Panel **(A)** shows the critical connectivity p^*^_L_ vs. the layer size ω for different coupling strengths (ϵ = {0.05 mV (red), 0.1 mV (cyan), 0.125 mV (green), 0.2 mV (blue), and 0.4 mV (black)}) and panel **(B)** shows p^*^_L_ vs. the coupling strength ϵ for different layer sizes (ω = {50 (red), 100 (cyan), 150 (green), 200 (blue), and 400 (black)}). In the main panels we use a logarithmic scale, the insets have a linear scale. The squares indicate the connectivity above which a synchronous pulse propagates from the 1st to the 20th layer of a FFN in at least 50% of *n* = 30 trials. The critical connectivity given by Equation (32) (solid lines) with *x*_0_ = x^*^_0_ (cf. Equation 37) is in good agreement with computer simulations. As predicted p^*^_L_ ∝ (ϵω)^−1^ and the proportionality factor 1/λ is well approximated by the estimate 1/λ^*^ derived in Equation (30).

For *x*_0_ = x^*^_0_ the bifurcation diagram near the bifurcation point is well approximated already for *k* = 2 (cf. Figure 2) and Equation (30) provides a good estimate of the critical connectivity p^*^_L_ (cf. Figure 3).

#### 3.1.3. Influence of external network

In the previous section we derived an iterated map for the average group size (cf. Equation 13) and an approximation for the critical connectivity p^*^_L_ (cf. Equations 30 and 32) that marks the transition from FFNs which do not support propagation of synchrony to FFNs that do. In this section we focus on the robustness of our results. How does the critical connectivity change with the layout of the external network? For which parameter range does the estimate of the critical connectivity (given by Equations 30 and 32) yield reasonable results?

The derivation was based on the assumption that the ground state dynamics of the neurons of the FFN is completely determined by the external inputs. This assumption holds if the spontaneous firing rate ν of the neurons and/or the coupling strengths ϵ and/or the connectivity *p* are sufficiently small. We will generalize our approach and show how the impact of preceding layers on a layer's ground state can be taken into account. Thereafter we will compare the results with computer simulations, identify the regions in parameter space for which the derived approximations hold and discuss deviations between direct numerical simulations and analytics.

The first layer of an FFN receives inputs only from the external network and according to Equations (6, 7) the mean μ_1_ and standard deviation σ_1_ of its input is

(38) $$\mu_{1}=I_{0}$$

(39) $$\sigma_{1}=\epsilon^{\text{ext}}\sqrt{2\tau^{\text{m}}\nu^{\text{ext}}},$$

as assumed in the previous section. All following layers receive external inputs and spikes from their preceding layer(s). The mean μ_*n*_ and standard deviation σ_*n*_ of the input to neurons of the *n*th layer (with *n* ≥ 2) reads (cf. Equations 6 and 7)

(40) $$\mu_{n}=I_{0}+\tau^{\text{m}}p\omega \nu_{n-1}\epsilon$$

(41) $$\sigma_{n}=\sqrt{2\nu^{\text{ext}}\tau^{\text{m}}{\left(\epsilon^{\text{ext}}\right)}^{2}+p\omega \nu_{n-1}\tau^{\text{m}}\epsilon^{2}}.$$

Here we denote the spontaneous firing rate (in the absence of synchrony) of neurons of the (*n* − 1)th layer by ν_*n* − 1_. It is given by Equation (9) as

(42) $$\nu_{n-1}=\frac{1}{\sqrt{\pi }\tau^{\text{m}}}\frac{\Theta -\mu_{n-1}}{\sigma_{n-1}}\exp\left[-{\left(\frac{\Theta -\mu_{n-1}}{\sigma_{n-1}}\right)}^{2}\right].$$

From layer to layer, the mean input, the standard deviation as well as the firing rate increase. For setups, where the ground state of the FFN is non-pathological, i.e., the firing rates of all layers are bounded, the additional corrections Δ*X*_*n*_ := *X*_*n*_ − *X*_*n* − 1_ for *X* ∈ {μ, σ, ν} decrease with *n*, and μ_*n*_, σ_*n*_ and ν_*n*_ saturate for sufficiently large *n*. Thus, μ_∞_ and σ_∞_ describe the input to the neurons of an infinitely long FFN and the single neurons of such an FFN spike with an average rate ν_∞_. Accordingly, replacing μ and σ by μ_∞_ and σ_∞_ in Equation (13) [where they appear as parameters of *p*_*f*_(·)] yields an iterated map for the average group size.

**Figure 4:**
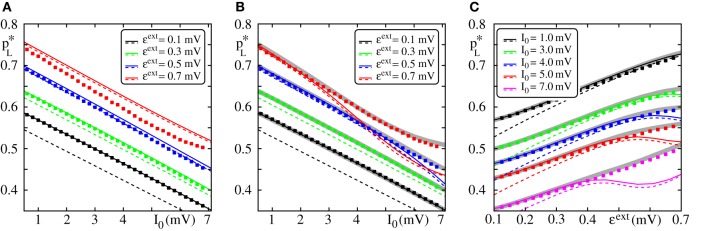
**Robustness of analytical estimates of the critical connectivity**. **(A–C)** We consider the critical connectivity p^*^_L_ of infinitely long FFNs, that are approximated by an FFN (*m* = 20, ω = 150, ϵ = 0.2 mV) with periodic boundary conditions in direct numerical simulations (markers), for different layouts of the external network. Panels **(A,B)** show p^*^_L_ vs. *I*_0_ for fixed ϵ^ext^ and panel **(C)** shows p^*^_L_ vs. ϵ^ext^ for fixed *I*_0_. The solid (colored) lines indicate the critical connectivity found by numerically determining the bifurcation point of the iterated map (Equation 13). In panel **(A)** we neglect the influence of previous layers on the ground state of a considered layer in the analytical computations [i.e., we use μ_1_ and σ_1_, cf. Equations (38) and (39)]. In **(B,C)** we employ corrections to account for their influence, cf. Equations (38–42). We show the third order correction, higher orders add only small modifications to the curves, but the numerical computations get more costly. The thick gray lines in **(B,C)** indicate the bifurcation point of the iterated map (Equation 13) with *P*_*V*_(*V*) derived from the diffusion approximation of leaky integrate-and-fire neuron dynamics with Poissonian input (Brunel and Hakim, 1999; Brunel, 2000). The dashed lines are the estimates of the critical connectivity given by Equations (30 and 32). Again, in panel **(A)** we neglect the influence of previous groups on the ground state, in panels **(B,C)** we use the third order correction. The estimates agree with the data from numerical simulations within the biologically relevant parameter range, where (1) the spontaneous spiking activity is low and (2) the distribution of membrane potentials is sufficiently broad. For further explanations see text (section 3.1.3).

In Figure 4, we compare the critical connectivity found by numerically determining the bifurcation point of the iterated map (Equation 13) (i.e., we determined the connectivity *p* for which the iterated map touches the diagonal; solid lines) with computer simulations of propagating synchrony (markers). To also cover scenarios, where the input from the preceding layer is not negligible, we consider infinitely long FFNs (then, the distribution of membrane potentials is equal in all layers). In computer simulations this can be approximated by a sufficiently long FFN with periodic boundary conditions, i.e., an FFN where the last layer connects to the first layer. For moderate external inputs, i.e., moderate *I*_0_ and ϵ^ext^, already the analytical results neglecting the influence of the preceding layers (using μ_1_ and σ_1_) agree well with computer simulations (cf. Figure 4A, solid lines). However, for large external inputs, i.e., large *I*_0_ and ϵ^ext^, the critical connectivity is overestimated. Here, the assumption that the distribution of membrane potentials is not influenced by the connectivity of the FFN does not hold. The additional input shifts the membrane potentials to higher values and consequently a lower connectivity is required for a propagation of a synchronous pulse. The corrections given by Equations (38–42) account for these deviations to some extent (cf. Figures 4B,C; solid lines), in particular for setups where the spontaneous firing rate is low. However, for very large *I*_0_ and ϵ^ext^, the critical connectivity is under-estimated. Here, the spontaneous firing rate is too high and the low-rate approximation, Equations (8–9), is not adequate to describe the system; the firing rate and thus the mean input from the previous layer are over-estimated. This becomes particularly clear in Figure 4C, where we show the critical connectivity as a function of the strength of the external inputs ϵ^ext^. For any given *I*_0_ (different colors), the critical connectivity for small ϵ^ext^ is well approximated; with increasing ϵ^ext^ the firing rate increases [α decreases and thus ν increases; cf. Equations (9 and 10)] and when the coupling strengths ϵ^ext^ exceed a *I*_0_-dependent threshold, the low-rate approximation becomes inapplicable.

Applying the methods in Brunel and Hakim (1999); Brunel (2000), the firing rate and the distribution of membrane potentials can be derived in diffusion approximation for states with higher spontaneous firing rates. Although most of the analytical considerations above are also applicable within this approximation, the determination of an optimal expansion point (cf. Equations 36 and 37) becomes more difficult and a closed form expression does not exist. However, the critical connectivity can be obtained by numerically determining the fixed points of the iterated map (Equation 13) and we find that it agrees with computer simulations for the entire considered range of *I*_0_ and ϵ^ext^, (cf. Figures 4B,C; gray lines).

Analogous to the approach presented above, corrections for the influence of preceding layers can be taken into account for the analytical estimate of the critical connectivity derived in the previous section (Equations 30 and 32). Replacing the connectivity *p* by the approximation p^*^_L_ = (λ^*^ϵω)^−1^ in Equations (40, 41) yields

(43) $$\mu_{n}=I_{0}+\tau^{\text{m}}/\lambda_{n-1}^{*}\nu_{n-1}$$

(44) $$\sigma_{n}=\sqrt{2\nu^{\text{ext}}\tau^{\text{m}}{\left(\epsilon^{\text{ext}}\right)}^{2}+\epsilon \nu_{n-1}\tau^{\text{m}}/\lambda_{n-1}^{*}}$$

where λ^*^_n − 1_ := λ^*^ (μ_*n* − 1_, σ_*n* − 1_) is given by Equation (30) and ν_*n* − 1_ = ν (μ_*n* − 1_, σ_*n* − 1_) is given by Equation (42). In Figure 4 we show the estimate of the critical connectivity p^*^_L_ = (λ^*^_n_ϵω)^−1^ (cf. Equation 32) using λ^*^_1_ (panel a; dashed line), i.e., neglecting the influence of the preceding layers, and using a higher correction order (panel b,c; dashed line: third order). For sufficiently large ϵ^ext^ the critical connectivity found by numerically determining the bifurcation point agrees with the analytical estimate given by Equation (32). As discussed above, the corrections Equations (43, 44) account for the deviations from the simulated data as long as the total spontaneous firing rate is sufficiently low. However, for small ϵ^ext^ the critical connectivity is under-estimated. Here, the standard deviation of the inputs (cf. Equation 7) is low, such that the distribution of membrane potentials *P*_*V*_(*V*) is narrow [for ϵ^ext^ → 0: *P*_*V*_(*V*) → δ(*V* − μ); cf. Equation (8)], the spiking probability of one neuron, *p*_*f*_(·), increases steeply in a small interval [for ϵ^ext^ → 0: *p*_*f*_(*x*) → Θ (*x* − μ); cf. Equation (8)] and thus the approximation of *p*_*f*_(·) by the leading terms of a Taylor expansion is not sufficiently accurate.

However, in the biologically plausible parameter regime, where the firing rates are small and the distribution of membrane potentials is broad, the critical connectivity is approximated well by Equation (32) together with Equation (30) (defining λ^*^), Equation (37) (defining x^*^_0_) and the corrections that account for the influence of the preceding layers, Equations (43, 44).

#### 3.1.4. Characteristics of propagating synchronous pulses

In the previous sections, we have shown that a synchronous pulse may propagate along a diluted FFN. In this section we study the characteristics and properties of a propagating synchronous signal. We consider them at the transition to stable propagation, p^*^_L_, because there they depend only weakly on the network setup. How large is the fraction of neurons that participate in propagating synchrony? How does this fraction depend on the network setup?

To answer such questions, we consider the effect of a propagation synchronous pulse on the single layers in the network, as a measure for the effective pulse size. In other words, we consider the mean input μ_*L*_ a neuron receives from the preceding layer if a synchronous pulse propagates along the FFN at the critical connectivity p^*^_L_. It is given by the product of the connection probability p^*^_L_, the connection strength ϵ and the average size of a propagating synchronous signal γ_*L*_; using Equations (27) and (29) yields

(45) $$\mu_{L}=\gamma_{L}p_{L}^{*}\epsilon =\frac{P_{V}(\Theta -x_{0}^{*})+P_{V}^{\left(1\right)}(\Theta -x_{0}^{*})x_{0}^{*}-\lambda^{*}}{P_{V}^{\left(1\right)}(\Theta -x_{0}^{*})}$$

and after inserting λ^*^ as given by Equation (30),

(46) $$\mu_{L}=\sqrt{\frac{x_{0}^{*}\left(2P_{V}(\Theta -x_{0}^{*})+x_{0}^{*}P_{V}^{\left(1\right)}\left(\Theta -x_{0}^{*}\right)\right)-2p_{f}\left(x_{0}^{*}\right)}{P_{V}^{\left(1\right)}\left(\Theta -x_{0}^{*}\right)}.}\;\;\;\;\;$$

**Figure 5:**
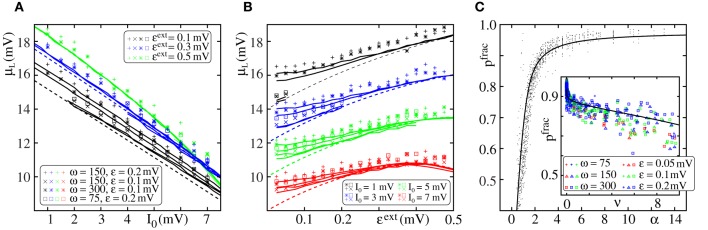
**Properties of propagating synchronous pulses at the transition from the no-propagation to the propagation regime**. Panels **(A,B)** show the mean input μ_*L*_ that a layer receives due to a propagating synchronous pulse in the preceding layer. μ_*L*_ measures the effective pulse size (the impact of a propagating synchronous pulse) and is mainly determined by the external inputs rather than by the setup of the FFN. In **(A)** the variance of the external input (measured by ϵ^ext^) is fixed and μ_*L*_ is plotted vs. *I*_0_; in **(B)** the mean external input *I*_0_ is fixed and μ_*L*_ is plotted vs. ϵ^ext^. The markers indicate μ_*L*_ for FFNs of different sizes [ω and ϵ are given by the legend in **(A)**] obtained by numerical simulations of propagating synchrony. The dashed lines shows the approximation of μ_*L*_ given by Equation (46) (which is independent of ω and ϵ); the solid lines indicate μ_*L*_ = p^*^_L_*G*_2_ϵ; values of p^*^_L_ and *G*_2_ are found semi-analytically, by numerically identifying the bifurcation point of the analytically derived iterated map (Equation 13) for the different network setups (both analytical estimates are corrected for the influence of inputs from the preceding layer up to the first order). Panel **(C)** shows the fraction *p*^frac^ of neurons in a layer that participate in the propagation of a synchronous signal vs. α [(Equation 10); main panel] and vs. the spontaneous firing rate ν (inset). Data from different network setups are plotted without distinction as black dots in the main panel and with distinction by different colors and symbols in the inset (see legend); Simulations are repeated for different layouts of the external network (*I*_0_ ∈ {1, 3, …, 11} mV; ϵ^ext^ ∈ {0.1, 0.125, …, 1.0} mV). The solid lines indicate *p*_*f*_ (μ_*L*_) = *f*_*p*_ (α) as given by Equation (53). The layer size ω as well as the coupling strength ϵ influence *p*^frac^ only weakly. *p*^frac^ depends on the network setup mainly through α or, equivalently, through ν (cf. Equation 9): Measurement values from different network setups largely collapse to the graph of the function *p*_*f*_ (μ_*L*_) = *f*_*p*_ (α). For further explanations see text (section 3.1.4).

According to Equation (46) the average input μ_*L*_ to the neurons due to a propagation of a synchronous pulse is independent of the layer size ω and coupling strength ϵ. For setups with moderate external inputs (i.e., inputs of the preceding layer influence the neurons' ground state only weakly; see also section 3.1.3) the distribution of membrane potentials *P*_*V*_(·) (cf. Equation 8), the firing probability of single neurons *p*_*f*_(·) (cf. Equation 14) as well as the expansion point (deflection point of *P*_*V*_(·); cf. Equation 37)

(47) $$x_{0}^{*}=\Theta -I_{0}+\epsilon^{\text{ext}}\sqrt{\tau^{\text{m}}\nu^{\text{ext}}}$$

are fully determined by the external inputs (*I*_0_, ν^ext^ and ϵ^ext^). Figures 5A,B illustrates the dependence of μ_*L*_ on the layout of the external network and the FFN: as expected from our analytical considerations, the dependence on the layer size and coupling strength is weak when *I*_0_ and ϵ^ext^ are kept fixed. With increasing mean of the external input (*I*_0_) the distribution of membrane potentials *P*_*V*_(*V*) is shifted toward the threshold Θ, such that it is more likely to find the membrane potential of the neurons near the threshold and the critical connectivity decreases (cf. also Figures 4A,B). Naturally this implies a decreasing average input μ_*L*_ at p^*^_L_, which is shown in Figure 5A for different external couplings ϵ^ext^ and parameters of the FFN. Increasing the external coupling strength ϵ^ext^ (and with it the variance of the external input) causes a broadening of the distribution of membrane potentials; the membrane potentials of some neurons are shifted toward the threshold and the membrane potentials of other neurons are shifted away from it. If the fraction of neurons that participate in the propagation of the synchronous pulse is large, this implies an increasing critical connectivity (Figure 5B; cf. also Figure 4C).

The spiking probability of a single neuron due to the mean input μ_*L*_ equals the average fraction *p*^frac^ of neurons of one layer that participate in a propagating synchronous pulse,

(48) $$p^{\text{frac}}=\frac{\gamma_{L}}{\omega }=p_{f}\left(\mu_{L}\right).$$

Interestingly, in the considered regime of low spiking rates and sufficiently broad distribution of membrane potentials, where the approximations given in section 3.1.1 are applicable, *p*^frac^ depends on the setup of the external inputs only via the quotient $\alpha =\frac{\Theta -\mu }{\sigma }$ (cf. Equation 10), or, equivalently, on the spontaneous firing rate ν of the neurons (cf. Equation 9). This can be shown by combining Equations (8, 37) and (Equation 46),

(49) $$\mu_{L}=\sigma {\left(\frac{e\pi }{2}\right)}^{1/4}{\left[\frac{\left(\sqrt{2}+2\alpha \right)\left(3+\sqrt{2}\alpha \right)}{2\sqrt{e\pi }}-\text{Erf}\left(\frac{1}{\sqrt{2}}\right)-\text{Erf}\left(\alpha \right)\right]}^{1/2}$$

(50) $$=:\sigma f_{\mu }(\alpha )$$

such that

(51) $$p_{f}(\mu_{L})=\frac{1}{2}\left[\text{Erf}\left(\frac{\Theta -\mu }{\sigma }\right)+\text{Erf}\left(\frac{\mu_{L}-\Theta +\mu }{\sigma }\right)\right]$$

(52) $$=\frac{1}{2}\left[\text{Erf}\left(\alpha \right)+\text{Erf}\left(\frac{\mu_{L}}{\sigma }-\alpha \right)\right]$$

(53) $$=\frac{1}{2}\left[\text{Erf}\left(\alpha \right)+\text{Erf}\left(f_{\mu }(\alpha )-\alpha \right)\right]=:f_{p}(\alpha ).$$

In Figure 5C we compare the above predictions with direct numerical simulations: For different layer sizes ω, coupling strengths ϵ and layouts of the external networks (i.e., different values of *I*_0_ and ϵ^ext^), we detect whether propagation of a synchronous pulse is possible and if so, we numerically determine the average fraction of participating neurons as well as the spontaneous firing frequency. We find that indeed the size of the synchronous pulse is determined essentially by the quotient $\alpha =\frac{\Theta -\mu }{\sigma }$ and Equation (53) is a reasonable estimate of the average fraction of neurons spiking in each layer. With increasing α the fraction of participating neurons increases, it thus decreases with spontaneous firing rate ν see Figure 5C. For FFNs with low spontaneous spiking frequency almost all neurons of a layer participate in the propagation of a synchronous pulse.

### 3.2. FFNs with non-linear dendrites

In this section, we investigate propagation of synchrony mediated by dendritic non-linearities. Although the mechanism underlying the propagation is generally related to that in linear networks, the discontinuities introduced by non-additive dendritic interactions prevent a similar analytical approach. In the first part of this section, we thus derive analytical estimates for the critical connectivity p^*^_NL_ in non-linearly coupled networks based on a self-consistency approach (see also Jahnke et al., 2012). In the second part, we study the transition from propagation of synchrony mediated by linear dendrites to propagation of synchrony mediated by non-additive dendritic interactions upon increasing the degree of non-linearity in the networks. In the last part, we evaluate the robustness of the analytical estimates with respect to the layout of the external network.

#### 3.2.1. Analytical derivation of critical connectivity

Neurons with non-additive dendritic interaction process excitatory input by a non-linear dendritic modulation function σ_*NL*_ (see section 2.1), i.e., synchronous inputs that exceed the dendritic threshold Θ_*b*_ are amplified to an effective input of size κ (cf. Equation 4). Therefore the spiking probability of a single neuron due to a synchronous input of strength *x, p*_*f*_(σ_*NL*_(*x*)), is discontinuous and an approach based on expansion of *p*_*f*_(·) is inappropriate. To derive an analytical expression for the critical connectivity p^*^_NL_ in FFNs incorporating dendritic non-linearities, we consider the (average) fraction of neurons of one layer, *p*_γ_, that receive an input *x* larger than the dendritic threshold, *x* ≥ Θ_*b*_, due to the propagating synchronous pulse. If there is a stable (stationary) propagation of synchrony established, *p*_γ_ is constant throughout the layers, which allows us to formulate a self-consistency equation. The basic derivations have been published recently (Jahnke et al., 2012) and will be briefly reviewed in the following for the readers convenience.

For sufficiently small dendritic thresholds Θ_*b*_ and sufficiently large κ, the spiking probability of a neuron due to a sub-threshold input is small compared to the spiking probability of a supra-threshold input. Therefore, we approximate the spiking probability of a single neuron in response to a synchronous input of strength *x* by

(54) $$p_{f}\left(\sigma_{NL}(x)\right)=\{\begin{array}{ll} p_{f}\left(\kappa \right) & \text{if }x\geq \Theta_{b}\\ 0 & \text{otherwise} \end{array},$$

i.e., we assume that somatic spikes due to the synchronous pulse are exclusively generated by dendritically enhanced inputs. We denote the fraction of neurons that receive a dendritic spike by *p*_γ_. This may be considered as constant throughout the different layers if stable propagation of synchrony is enabled. Then the probability that a neuron receives exactly *k* inputs from the preceding layer follows a binomial distribution *k* ~ *B*(ω, *p*_γ_*p*_*f*_ (κ) *p*), where *p*_γ_*p*_*f*_ (κ) *p* is the probability that (1) a neuron of the preceding layer receives a supra-threshold input (*p*_γ_), (2) a somatic spike is elicited by that input (*p*_*f*_ (κ)) and there is a connection from this spiking neuron to the considered neuron of the following layer (*p*). So we can formulate the self-consistency equation for *p*_γ_,

(55) $$p_{\gamma }=\sum_{k=\left\lceil \Theta_{b}/\epsilon \right\rceil }^{\omega }\left(\begin{array}{c} \omega \\ k \end{array}\right){\left(p_{\gamma }p_{f}\left(\kappa \right)p\right)}^{k}{\left(1-p_{\gamma }p_{f}\left(\kappa \right)p\right)}^{\omega -k}.$$

To solve Equation (55) we approximate the binomial distribution by a Gaussian distribution with mean δ := ω *p*_γ_*pp*_*f*_ (κ) and standard deviation $\sigma_{\delta }:=\sqrt{\delta (1-p_{\gamma }pp_{f}\left(\kappa \right))}$, which yields

(56) $$p_{\gamma }=\frac{1}{2}\left[1+\text{Erf}\left(\frac{n}{\sqrt{2}}\right)\right],$$

where we defined

(57) $$n:\text{​}=\frac{\delta -\Theta_{b}/\epsilon }{\sigma_{\delta }}$$

(58) $$=\frac{\omega p_{\gamma }pp_{f}\left(\kappa \right)-\Theta_{b}/\epsilon }{\sqrt{\omega p_{\gamma }pp_{f}\left(\kappa \right)(1-p_{\gamma }pp_{f}\left(\kappa \right))}}$$

as the difference between the average number of inputs (δ) and the number of inputs needed to reach the dendritic threshold (Θ_*b*_/ϵ) normalized by the standard deviation of the number of inputs (σ_δ_). Solving definition (Equation 58) for *p* and replacing *p*_γ_ by Equation (56) yields

(59) $$p_{NL}=\frac{n^{2}\epsilon +2\Theta_{b}+n\sqrt{n^{2}\epsilon^{2}+4\Theta_{b}\left(\epsilon -\frac{\Theta_{b}}{\omega }\right)}}{p_{f}(\kappa )\epsilon (n^{2}+\omega )\left(1+Erf\left(\frac{n}{\sqrt{2}}\right)\right)},$$

which is the connectivity *p*_*NL*_ where stable propagation of synchrony with some given *n* (or, equivalently, some given *p*_γ_; cf. Equation 56) is established. We note that a propagation of synchrony mediated by dendritic spikes requires

(60) $$\epsilon \omega >\Theta_{b}$$

(otherwise even the input caused by a synchronized spiking of all neurons of a layer in a fully connected FFN (*p* = 1) is not sufficient to reach the dendritic threshold Θ_*b*_).

For parameters fulfilling the inequality (Equation 60), *p*_*NL*_(*n*) has a global minimum (see Appendix) and the critical connectivity p^*^_NL_, again defined as the smallest connectivity that allows for a stable propagation of synchrony, matches that global minimum: any connectivity *p*_*NL*_ above the minimal connectivity p^*^_NL_ has two preimages *n*_1_ and *n*_2_ corresponding to the both non-trivial fixed points *G*_1_ and *G*_2_ of the iterated map for the average group size (cf. Figure 1 and section 2.4). However, there exists smaller connectivities for which a stationary propagation can be established. At the global minima p^*^_NL_ both preimages *n*_1_ and *n*_2_ collapse to n^*^ = *n*_1_ = *n*_2_ and correspond to the fixed point *G* = *G*_1_ = *G*_2_ of the iterated map at the bifurcation point of the tangent bifurcation. Here the transition from the regime where no propagation of synchrony is possible to the regime where a propagation of synchrony is enabled takes place. For *p*_*NL*_ smaller than p^*^_NL_ there are no preimages (i.e., a stationary propagation of synchrony mediated by non-additive dendritic interactions cannot be established); this scenario correspond to the absence of the non-trivial fixed points of the iterated map for connectivities below the tangent bifurcation.

In the following we will obtain the minima of *p*_*NL*_ (i.e., the critical connectivity p^*^_NL_) in the limit of large layer sizes ω and small coupling strength ϵ. We first derive an approximation of Equation (59) (cf. Equation 62), determine the validity range of this approximation (cf. Equation 69) and finally obtain an estimate for the critical connectivity (cf. Equation 71). As before, we fix the maximal input ϵω to each neuron to preserve the network state and expand Equation (59) in a power series around ϵ → 0 and ω → ∞. Considering the leading terms yields

(61) $$p_{NL}\approx p_{NL,a}:=\frac{2\Theta_{b}}{p_{f}\left(\kappa \right)\epsilon \omega }\frac{1+n\sqrt{\frac{\epsilon }{\Theta_{b}}-\frac{1}{\omega }}}{1+\text{Erf}\left(\frac{n}{\sqrt{2}}\right)}.$$

Further a propagation mediated by dendritic spikes (as introduced above) requires that the layer size ω and the coupling strength ϵ are sufficiently large such that a sufficiently large fraction of neurons of each layer receive a total input larger than the dendritic threshold Θ_*b*_. In particular for diluted FFNs, this requirement translates to ϵω » Θ_*b*_ and Equation (61) simplifies further to

(62) $$p_{NL,b}:=\frac{2\Theta_{b}}{p_{f}\left(\kappa \right)\epsilon \omega }\frac{1+n\sqrt{\frac{\epsilon }{\Theta_{b}}}}{1+\text{Erf}\left(\frac{n}{\sqrt{2}}\right)}.$$

**Figure 6:**
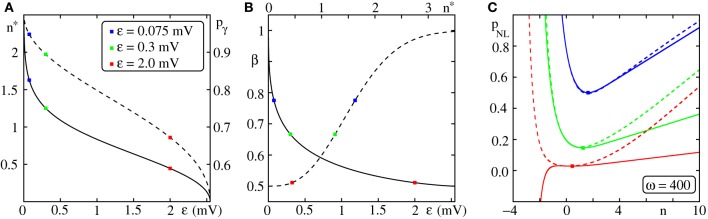
**Determining the critical connectivity in FFNs with non-additive dendritic interactions. (A)** For a given setup, i.e., for a given dendritic threshold Θ_*b*_ and coupling strength $\epsilon <\frac{2\Theta_{b}}{\pi }$, the corresponding n^*^ (or equivalently *p*_γ_; cf. Equation 56) is found by Equation (64). The solid line indicates n^*^ vs. ϵ (left vertical scale), the dashed line *p*_γ_ vs. ϵ (right vertical scale) and the markers n^*^(ϵ) for ϵ = {0.075, 0.3, 2.0} mV (see legend). [Here, the dendritic threshold is Θ_*b*_ = 4 mV, such that the estimate (Equation 64) is valid within the range ϵ ∈ (0, 2.55] mV; Equation (69)] **(B)** Knowing n^*^ allows to evaluate $\beta \left(\frac{\Theta_{b}}{\epsilon }\right)\in \left[\frac{1}{2},1\right]$ according to Equation (70). Panel **(B)** shows β (cf. Equation 70) vs. ϵ (solid line, lower horizontal axis) and β vs. n^*^ (dashed line, upper horizontal axis), respectively. **(C)** Finally, the critical connectivity p^*^_NL_ is obtained by Equation (71) which depends on $\beta \left(\frac{\Theta_{b}}{\epsilon }\right)$. Panel **(C)** shows the connectivity *p*_*NL*_[dashed; Equation (59)] and its approximation *p*_*NL, b*_ [solid; Equation (63)] vs. *n*; for ϵ ∈ (0, ϵ^*max*^], *p*_*NL, b*_ has a local minimum which agrees with the global minimum of *p*_*NL*_. The markers indicate the critical connectivity p^*^_NL_ obtained by the procedure described in **(A)** and **(B)**. For further explanations see text (section 3.2.1).

Whereas *p*_*NL*_ has always a global minimum for ϵω > Θ_*b*_, this does not hold for the approximation *p*_*NL, b*_, e.g., (cf. also Figure 6C)

(63) $$\underset{n\to -\infty }{\lim}\left(p_{NL,b}\right)=-\infty .$$

However, we will now show that *p*_*NL, b*_ has a (local) minimum if (and only if) $\epsilon \in \left(0,\;\frac{2\Theta_{b}}{\pi }\right]$ which approximates the global minimum of *p*_*NL*_ and therefore serve as an estimate for the critical connectivity. Starting with ${\frac{dp_{NL,\;b}(n)}{dn}|}_{n=n^{*}}=0$ yields

(64) $$\sqrt{\frac{\Theta_{b}}{\epsilon }}=\sqrt{\frac{\pi }{2}}\exp\left(\frac{n^{{*}^{2}}}{2}\right)\left(1+\text{Erf}\left(\frac{n^{*}}{\sqrt{2}}\right)\right)-n^{*}=:f\left(n^{*}\right),$$

and n^*^ specifies the extremum of *p*_*NL, b*_(*n*). The second derivative of *p*_*NL, b*_(*n*) at the extremum n^*^ given by Equation (64) satisfies

(65) $${\frac{dp_{NL,b}^{2}}{dn^{2}}|}_{n=n^{*}}=\frac{2n^{*}\sqrt{\frac{\Theta_{b}}{\epsilon }}}{p_{f}\left(\kappa \right)\omega \left(1+\text{Erf}\left[\frac{n^{*}}{\sqrt{2}}\right]\right)}>0$$

if n^*^ > 0 such that the extremum actually is a minimum. Taken together, for a given setup, i.e., for given dendritic threshold Θ_*b*_ and coupling strength ϵ, the transcendent Equation (64) defines n^*^ which maximizes or minimize *p*_*NL, b*_(*n*) and if additionally n^*^ > 0 the extremum *p*_*NL, b*_(n^*^) is a minimum.

Differentiating the right hand side of Equation (64),

(66) $$\frac{df(n^{*})}{dn^{*}}=n^{*}\cdot e^{\frac{n^{*2}}{2}}\sqrt{\frac{\pi }{2}}\left(1+\text{Erf}\left[\frac{n^{*}}{\sqrt{2}}\right]\right)$$

(67) $$\frac{d^{2}f(n^{*})}{dn^{*2}}=n^{*}+\left(1+n^{*2}\right)e^{\frac{n^{*2}}{2}}\sqrt{\frac{\pi }{2}}\left(1+\text{Erf}\left[\frac{n^{*}}{\sqrt{2}}\right]\right),\;$$

shows that *f*(n^*^) (as defined in Equation 64) is (1) minimal for n^*^ = 0 and (2) monotonically increasing for n^*^ > 0; according to Equation (64) the minimum n^*^ = 0 corresponds to

(68) $$\epsilon^{\text{max}}:=\frac{\Theta_{b}}{{\left[f(0)\right]}^{2}}=\frac{2\Theta_{b}}{\pi }\approx 0.64\Theta_{b}.$$

The left hand side of Equation (64), i.e., $\sqrt{\Theta_{b}/\epsilon }$, is monotonically decreasing with ϵ from infinity to zero. Thus Equation (64) has a solution for any

(69) $$\epsilon \in \left(0,\epsilon^{\text{max}}\right]=\left(0,\frac{2\Theta_{b}}{\pi }\right]$$

and p^*^_NL_ := p^*^_NL, b_(n^*^) is the (local) minimum of Equation (62) and provides an estimate for the critical connectivity, the (global) minimum of Equation (59).

For better readability we define the function β(·),

(70) $$\beta \left(\frac{\Theta_{b}}{\epsilon }\right):=\frac{1}{2}\left(1+\text{Erf}\left[\frac{n^{*}}{\sqrt{2}}\right]\right)-n^{*}\frac{e^{-\frac{n^{{*}^{2}}}{2}}}{\sqrt{2\pi }},$$

where $n^{*}=n^{*}\left(\frac{\Theta_{b}}{\epsilon }\right)$ as given by Equation (64). We note that $\beta \left(\frac{\Theta_{b}}{\epsilon }\right)$ can also be considered as a function of n^*^. By combining Equations (62), (64), and (70) we obtain the critical connectivity

(71) $$p_{NL}^{*}=\frac{\Theta_{b}}{p_{f}\left(\kappa \right)\epsilon \omega }\cdot \frac{1}{\beta \left(\frac{\Theta_{b}}{\epsilon }\right)}.$$

The function β(·) itself is monotonically decreasing with ϵ in the validity range ϵ ∈ (0, ϵ^max^] of the above approximation: within this interval n^*^ > 0 and $\frac{d}{dn^{*}}f\left(n^{*}\right)>0$ and thus the derivative

(72) $$\frac{d\beta }{d\epsilon }=\frac{d\beta }{dn^{*}}\cdot \frac{dn^{*}}{d\sqrt{\Theta_{b}/\epsilon }}\cdot \frac{d\sqrt{\Theta_{b}/\epsilon }}{d\epsilon }$$

(73) $$=-\frac{e^{-\frac{n^{*2}}{2}}n^{*2}}{\sqrt{2\pi }}\cdot {\left(\frac{df\left(n^{*}\right)}{dn^{*}}\right)}^{-1}\cdot \sqrt{\frac{\Theta_{b}}{4\epsilon^{3}}}$$

(74)   <  0.

Consequently β assumes its minimum

(75) $$\beta^{\text{min}}=\beta \left(n^{*}=0\right)=\frac{1}{2}$$

for $\epsilon =\epsilon^{\text{max}}=\frac{2\Theta_{b}}{\pi }$ and increases monotonically with decreasing ϵ against its asymptotic value

(76) $$\beta^{\text{max}}=\underset{n^{*}\to \infty }{\lim}\left[\frac{1}{2}\left(1+\text{Erf}\left[\frac{n^{*}}{\sqrt{2}}\right]\right)-n^{*}\frac{e^{-\frac{n^{{*}^{2}}}{2}}}{\sqrt{2\pi }}\right]=1.$$

Thus the critical connectivity is bounded by

(77) $$p^{0}:=\frac{\Theta_{b}}{p_{f}\left(\kappa \right)\epsilon \omega }\leq p_{NL}^{*}\leq 2\cdot \frac{\Theta_{b}}{p_{f}\left(\kappa \right)\epsilon \omega }=2\cdot p^{0}$$

and converges to the lower bound *p*^0^ for small ϵ and to its upper bound 2*p*^0^ for large ϵ.

**Figure 7:**
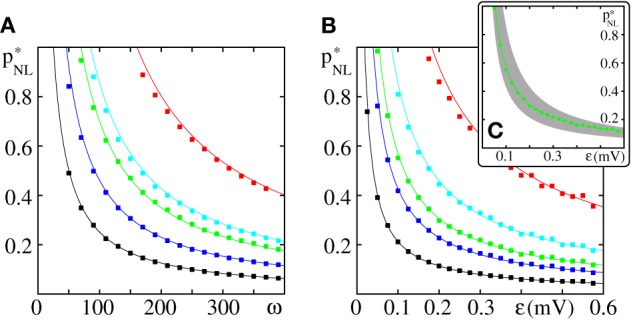
**Critical connectivity in FFNs with non-linear dendrites**. The panels show **(A)** the critical connectivity p^*^_NL_ vs. the layer size ω for different coupling strengths (ϵ = {0.05 mV (red), 0.1 mV (cyan), 0.125 mV (green), 0.2 mV (blue), and 0.4 mV (black)}) and **(B)** p^*^_NL_ vs. the coupling strength ϵ for different layer sizes (ω = {50 (red), 100 (cyan), 150 (green), 200 (blue), and 400 (black)}). The points indicate the minimal connectivity for which a synchronous pulse propagates from the first to the last layer in an FFN with *m* = 20 layers in at least 50% of *n* = 30 trials. The critical connectivity given by Equation (71) (solid lines) is in good agreement with the computer simulations. **(C)** The critical connectivity is confined to the interval p^*^_NL_ ∈ [*p*^0^, 2*p*^0^] [indicated by the gray area for ω = 150 (green), cf. Equation (77)] and approaches its lower bound for small ϵ and its upper bound for large ϵ. Like in linearly coupled networks the critical connectivity decays inversely proportional to layer size, p^*^_NL_ ∝ ω^−1^, (cf. also Figure 3), but the scaling with coupling strength is more complicated, $p_{NL}^{*}\propto \epsilon^{-1}\;\cdot \;1/\beta \left(\frac{\Theta_{b}}{\epsilon }\right)$; the factor $\beta \left(\frac{\Theta_{b}}{\epsilon }\right)\in \left[0.5,\;1\right]$ [cf. Equation (70) and Figure 6] measures the deviation from the algebraic decay (as found in linearly coupled networks). In this figure the parameters of the external network are fixed to *I*_0_ = 5 mV, ν^ext^ = 3 kHz, ϵ^ext^ = 0.5 mV.

In Figure 6 we visualize the determination of the critical connectivity (Equations 64, 70) and Equation (71). The critical connectivity obtained with the approach presented above agrees well with simulation data (cf. Figure 7).

#### 3.2.2. Transition from linear to non-linear propagation

In the previous section we derived analytical estimates for the critical connectivity p^*^_NL_ in FFNs with non-additive dendritic interactions; p^*^_NL_ is determined by (1) the setup of the FFN (i.e., the layer size ω and coupling strength ϵ; cf. Figure 7), (2) the parameters of the non-linear modulation function (i.e., the dendritic threshold Θ_*b*_ and enhancement level κ) and (3) the layout of the external network (i.e., the mean external input *I*_0_ and its variance, which is proportional to ϵ^ext^). In this section, we discuss the influence of the parameters of the non-linear modulation function and study the transition from a regime where propagation of synchrony is mediated by dendritically enhanced inputs to a regime where the majority of inputs is processed linearly.

In general, with increasing threshold Θ_*b*_ more and more inputs are needed to reach this threshold and consequently the critical connectivity p^*^_NL_ increases. If Θ_*b*_ exceeds μ_*L*_, which is the average input to the neurons if a synchronous pulse propagates in linearly coupled FFNs (cf. Equation 45 and Figure 5), propagation mediated by linearly processed spikes is enabled for lower connectivities than propagation mediated by dendritic non-linearities. In this regime the linearly summed inputs (for *p* = p^*^_L_) are sufficient to maintain propagation of synchrony, but are not sufficient to cross the dendritic threshold. Increasing Θ_*b*_ even further has no influence on the critical connectivity p^*^_NL_, here a propagation of synchrony is possible for *p* ≥ p^*^_L_ as discussed in section 3.1.

**Figure 8:**
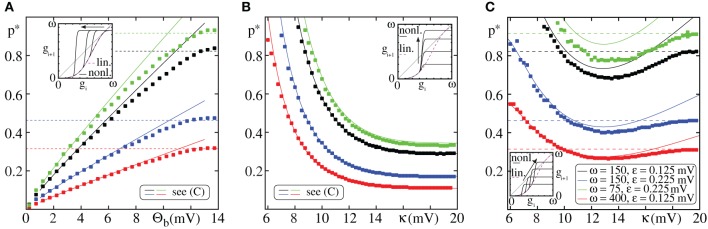
**Transition from linear to non-linear propagation**. The figure shows the critical connectivity p^*^_NL_ vs. the parameters of the non-linear modulation function σ_*NL*_ (cf. Equation 4) for different network setups (color code, see **(C)**). The lines are the theoretical predictions for p^*^_NL_ [solid, Equation (71)] and p^*^_L_ [dashed, Equation (32)]. The markers indicate the minimal connectivity for which a synchronous pulse propagates from the first to the last layer in an FFN (*I*_0_ = 5 mV, ν^ext^ = 3 kHz, ϵ^ext^ = 0.5 mV) with *m* = 20 layers in at least 50% of *n* = 30 trials. The insets illustrate the effect of changing Θ_*b*_ and κ on the iterated map, cf. Equation (13), where connectivity is kept constant. **(A)** Critical connectivity vs. dendritic threshold Θ_*b*_ for constant enhancement level κ = μ_*L*_ ≈ 13.7 mV (cf. Equation 50). If the dendritic threshold Θ_*b*_ is sufficiently small such that *p*_*f*_ (Θ_*b*_) « *p*_*f*_ (κ) (cf. Equation 54), the propagation of synchrony is mainly mediated by non-linear enhanced inputs and the critical connectivity can be estimated by Equation (71). For large Θ_*b*_ the probability that an input from the preceding layer exceeds the dendritic threshold is very low, propagation of synchrony is mainly mediated by linearly processed inputs and the critical connectivity is given by Equation (32). Between these scenarios (for moderate Θ_*b*_) there is a “transition regime,” where linear and non-linear propagation mix [similarly in **(C)**]. **(B)** Critical connectivity vs. enhancement level κ for constant threshold Θ_*b*_ = 4 mV. For small enhancement levels κ the (maximal) spiking probability of a single neuron, *p*_*f*_ (κ), is small and thus the critical connectivity p^*^_NL_ is large. With increasing κ, *p*_*f*_ (κ) increases and thus p^*^_NL_ decreases; for large κ, *p*_*f*_ (κ) → 1 (a neuron will almost surely spike upon the receipt of a non-linearly enhanced pre-synaptic input) and the critical connectivity saturates. **(C)** Critical connectivity vs. enhancement level κ for an additive enhancement by a constant Δ = κ − Θ_*b*_ = 4 mV. For further explanations see text (section 3.2.2).

We illustrate this transition from non-linear to linear propagation in Figure 8A: We start with large Θ_*b*_ = μ_*L*_ such that propagation is enabled for *p* ≈ p^*^_L_ and also set κ = μ_*L*_. In fact, the linear critical connectivity p^*^_L_ slightly under-estimates the observed critical connectivity p^*^_NL_ as it does not account for the saturation of the non-linear modulation function, i.e., for the cutoff σ_*NL*_(*x*) = κ of inputs *x* ≥ κ. With decreasing Θ_*b*_ the critical connectivity is substantially reduced and well approximated by Equation (71). Propagation of synchrony is now mainly mediated by dendritically enhanced inputs as described in section 3.2.1. The inset illustrates the impact of decreasing the dendritic threshold Θ_*b*_ on the iterated map. Initially, for Θ_*b*_ = μ_*L*_ = κ, the iterated map for linearly coupled and non-linearly coupled FFNs is similar; with decreasing Θ_*b*_ the jump like rise in the iterated map is shifted to lower group sizes and consequently the bifurcation point is shifted to lower connectivities.

The non-linear modulation function σ_*NL*_(·) (cf. Equation 4) saturates for strong inputs, thus the enhancement level κ defines the maximal (effective) input to a neuron and *p*_*f*_ (κ) is an upper bound for the spiking probability of any neuron in response to incoming inputs. This implies that in contrast to linearly coupled FFNs, the average size of a propagating synchronous pulse, γ_*NL*_, given by the product of the probability of a neuron receiving sufficiently strong input to reach the dendritic threshold (*p*_γ_; cf. Equation 56), the spiking probability due to that input [*p*_*f*_ (κ)] and the layer size ω, is bounded from above by

(78) $$\gamma_{NL}=p_{\gamma }p_{f}\left(\kappa \right)\omega \leq \omega p_{f}\left(\kappa \right)=:\gamma^{\text{max}}.$$

This bound decrease with decreasing κ as illustrated by Figure 8B (inset), where we compare the iterated maps for different values of κ. *p*_*f*_ (κ) also influences the critical connectivity p^*^_NL_ (cf. Equation 71): For small κ the spiking probability *p*_*f*_ (κ) is low and thus p^*^_NL_ is large (it may even exceed p^*^_L_). With increasing κ also *p*_*f*_ (κ) increases and consequently the critical connectivity p^*^_NL_ decreases; for very large κ the spiking probability *p*_*f*_ (κ) approaches 1 (cf. Equation 14) and p^*^_NL_ saturates (cf. Figure 8B).

In Figure 8C we show the critical connectivity for an additive enhancement by a constant Δ, i.e., inputs exceeding the dendritic threshold Θ_*b*_ are increased by the constant value Δ = κ − Θ_*b*_. For small κ the critical connectivity p^*^_NL_ is relatively large and may exceed p^*^_L_ due to the low saturation level of the non-linear modulation function σ_*NL*_(·) (cf. also Figure 8B). As mentioned above, with increasing κ, also *p*_*f*_ (κ) increases and the critical connectivity p^*^_NL_ decreases. However, for large κ and thus large dendritic threshold Θ_*b*_ propagation of synchrony mediated by linearly processed spikes is possible for lower connectivities than propagation mediated by dendritic non-linearities. Consequently, p^*^_NL_ converges toward p^*^_L_ (cf. also Figure 8A).

#### 3.2.3. Influence of external network

In section 3.2.1 we derived an estimate of the critical connectivity p^*^_NL_ for FFNs with non-additive dendritic interactions. So far we discussed the influence of the setup of the FFN (layer size ω and coupling strength ϵ) as well as the parameters of the non-linear modulation function σ_*NL*_ (dendritic threshold Θ_*b*_ and enhancement level κ). In the current section, we focus on the remaining determining factor, the layout of the external network. How does the critical connectivity change with the mean external input *I*_0_ and external coupling strength ϵ^ext^ and how well are these changes covered by our analytics?

For the derivation of p^*^_NL_ we assumed that somatic spikes are elicited exclusively by dendritically enhanced inputs (cf. Equation 54) and thus the critical connectivity depends on the layout of the external network only via *p*_*f*_ (κ) (cf. also Equation 71), i.e., on the average spiking probability of a neuron receiving an input larger than the dendritic threshold *x* ≥ Θ_*b*_. For sufficiently small *p*_*f*_ (κ), p^*^_NL_ > 1 and propagation of synchrony is not possible. With increasing *p*_*f*_ (κ) the critical connectivity decreases and for *p*_*f*_ (κ) → 1 it converges to Θ_*b*_(ϵωβ[Θ_*b*_/ϵ])^−1^, independent of the external network.

In the regime of low spiking rates, changing the mean external input *I*_0_ simply shifts the distribution of membrane potentials *P*_*V*_(*V*) (which is a Gaussian distribution centered at *I*_0_; cf. Equation 8). Thus, with increasing *I*_0_, *p*_*f*_ (κ) increases and the critical connectivity p^*^_NL_ decreases.

**Figure 9:**
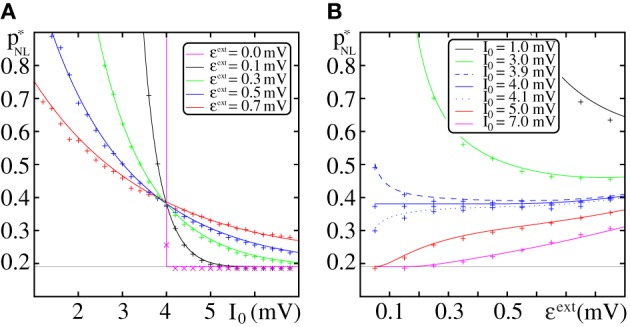
Dependence of the critical connectivity p^*^_NL_ on the layout of the external network. **(A,B)** The lines indicate the theoretical prediction for p^*^_NL_ given by Equation (71) and agree well with the data from direct numerical simulations (markers; FFN with ω = 150, ϵ = 0.2 mV, Θ_*b*_ = 4 mV, κ = 11 mV, *m* = 20). Panel **(A)** shows the critical connectivity vs. the mean external input *I*_0_ for fixed ϵ^ext^ and panel **(B)** shows the critical connectivity vs. ϵ^ext^ for fixed mean external input *I*_0_. The gray line indicates the minimal critical connectivity obtained for *p*_*f*_ (κ) = 1. With increasing mean (external) input *I*_0_ the distribution of membrane potentials *P*_*V*_(*V*) is shifted toward the somatic threshold Θ, thus the spiking probability *p*_*f*_ (κ) upon the reception of a non-linear enhanced input increases and the critical connectivity p^*^_NL_ decreases. For *I*_0_ = Θ − κ, *p*_*f*_ (κ) ≈ 0.5 (cf. Equation 80) and p^*^_NL_ is largely independent of the layout of the external network [blue solid line in **(B)**; cf. also **(A)** where all curves coincide]. Further explanations see text (section 3.2.3).

In Figure 9A we show the critical connectivity for different ϵ^ext^ [which determines the width of *P*_*V*_(*V*)] vs. the mean external input *I*_0_. For *I*_0_ = Θ − κ (such that the sum of a dendritically enhanced input and the center of the distribution of membrane potentials equals the somatic threshold Θ), *p*_*f*_ (κ) simplifies to

(79) $$p_{f}(\kappa )=\frac{1}{2}\left(\text{Erf}\left[\frac{\Theta -I_{0}}{\sigma }\right]+\text{Erf}\left[\frac{\kappa -\Theta +I_{0}}{\sigma }\right]\right)$$

(80) $$=\frac{1}{2}\text{Erf}\left(\frac{\Theta -I_{0}}{\sigma }\right)$$

and thus in the regime of low spiking rates, i.e., (Θ − *I*_0_)/σ » 1, *p*_*f*_ (κ) ≈ 0.5 independent of the width of the distribution of membrane potentials. Consequently, all curves for different ϵ^ext^ coincide at this point. For *I*_0_ > Θ − κ the majority of neurons (>50%) would spike upon receipt of a dendritically enhanced input. Thus *p*_*f*_ (κ) increases and therewith the critical connectivity decreases upon decreasing ϵ^ext^. In the limit of ϵ → 0, *P*_*V*_(*V*) converges toward a δ-distribution centered at *I*_0_ and *p*_*f*_ becomes a step-function

(81) $$p_{f}\left(\kappa \right)=\{\begin{array}{ll} 0 & \kappa <\Theta -I_{0}\\ 1 & \kappa \geq \Theta -I_{0} \end{array}$$

such that the critical connectivity is either constant and minimal for *I*_0_ ≥ Θ − κ or it diverges (no propagation possible) for *I*_0_ < Θ − κ (cf. Figure 9A; magenta curve).

In Figure 9B we illustrate the effect of changing ϵ^ext^ on the critical connectivity for constant *I*_0_. As discussed above for *I*_0_ = Θ − κ, *p*_*f*_ (κ) and thus p^*^_NL_ are rather independent of ϵ^ext^ and for *I*_0_ > Θ − κ the critical connectivity increases with ϵ^ext^. For *I*_0_ < Θ − κ an increase of the width of the distribution of membrane potentials shifts the membrane potential of more and more neurons toward the relevant interval [Θ − κ, Θ] and thus *p*_*f*_ (κ) increases and the critical connectivity p^*^_NL_ decreases.

For the derivation of p^*^_NL_ we have assumed that the ground state dynamics is essentially not influenced by the spontaneous activity of the FFN itself (i.e., μ = *I*_0_ and $\sigma =\epsilon^{\text{ext}}\sqrt{2\tau^{\text{m}}\nu^{\text{ext}}}$). As discussed in section 3.1.3, we can correct the results for such influences. However, since in non-linearly coupled FFNs the impact of (non-linearly enhanced) synchronous activity is much stronger than the impact of spontaneous activity (which is irregular and not amplified by non-additive dendritic interactions), we find that the deviations between the corrected and uncorrected version of p^*^_NL_ is negligible.

**Figure 10:**
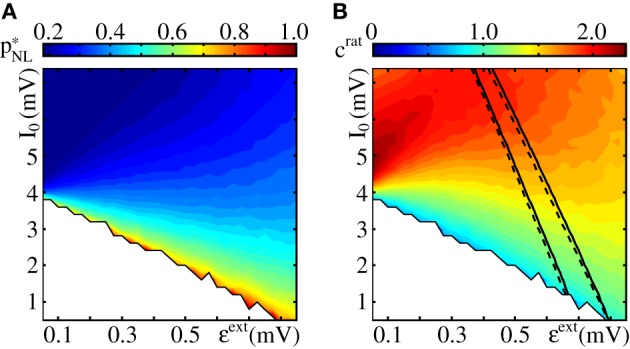
**Critical connectivity and reduction factor**. Panel **(A)** shows the critical connectivity obtained from simulations of an FFN (ω = 150, ϵ = 0.2 mV, *m* = 20) incorporating non-additive dendritic interactions (Θ_*b*_ = 4 mV, κ = 11 mV; see also Figure 9). Within the white area, propagation of synchrony is impossible because even for a fully coupled chain the input to the next layer (limited by the saturation of the non-linear modulation function and the layer size) is insufficient. Panel **(B)** shows the reduction factor *c*^rat^ (cf. Equation 83), the quotient between the critical connectivity in FFNs without and with non-additive dendritic interactions. The lines enclose the area for which the spontaneous firing is between ν ∈ [0.5, 1.5] Hz obtained from simulations (solid) and low-rate approximation (cf. Equation 9; dashed).

Finally, we compare the critical connectivity for networks with and without non-additive dendritic interactions: The factor

(82) $$c^{\text{rat}}:=\frac{p_{L}^{*}}{p_{NL}^{*}}=\frac{p_{f}\left(\kappa \right)}{\lambda \Theta_{b}}\beta \left(\frac{\Theta_{b}}{\epsilon }\right)$$

measures how much the connectivity within the FFN can be reduced by introducing non-additive dendritic interactions. It is independent of the layer size ω and becomes maximal in the limit of small coupling strengths ϵ as β (Θ_*b*_/ϵ) → β^max^ = 1 for ϵ → 0 (cf. Equation 76). It increases with decreasing Θ_*b*_ and increasing κ (see discussion in section 3.2.2). In Figure 10 we show the influence of the external network. As discussed above, for small *I*_0_, propagation of synchrony is not possible (the non-linear enhanced input is insufficient to elicit sufficiently many spikes in the layers of the FFN; white areas in Figure 10). With increasing *I*_0_, p^*^_NL_ decreases and *c*^rat^ increases.

### 3.3. Generalizations

In the final section we discuss generalizations of the methods and results we derived. Compared to biological neurons, our models have simplifications which enable the analytical treatment, but might be suspected to be influential on the final result. These simplifications are the homogeneous delay distribution, the simplified initiation and impact of dendritic spikes, the limit of short synaptic currents and the sub-threshold leaky integrate-and-fire dynamics. Here, we verify that our results generalize to biologically more detailed neurons without these simplifications. In particular, we show that the estimates for the critical connectivity hold. Further, we consider a qualitatively different dendritic interaction function which assumes that the saturation is incomplete, i.e., beyond a region of saturation the impact of larger inputs increases. We show that the tools developed in the article are still applicable and reveal a new phenomenon, the coexistence of linear and non-linear propagation of synchrony.

In the first part (section 3.3.1), we discuss the influence of inhomogeneous delay distribution and finite dendritic integration windows. In the second part (section 3.3.2), we consider the non-linear modulation function with incomplete saturation. Finally, we consider biologically more detailed neuron models (section 3.3.3).

#### 3.3.1. Heterogeneous delays

So far we considered FFNs with homogeneous delay distribution and dendritic modulation functions with integration window of zero length, i.e., only exactly synchronized inputs were possibly non-linearly amplified. Are these assumptions crucial for the obtained results? How does the critical connectivity change in the presence of heterogeneous delay distributions?

To answer this question, we consider synaptic delays τ_*kl*_ (specifying the synaptic delay between neuron *l* and *k*) uniformly drawn from

(83) $$\tau_{kl}\in \left[\tau -\frac{\Delta T}{2},\tau +\frac{\Delta T}{2}\right],$$

where τ is the mean delay. A direct consequence of heterogeneous delay distribution is that the spikes of the propagating synchronous signal are not simultaneous (i.e., exactly synchronized) anymore. To describe the system accurately one has to consider additionally to the size (*g*_*i*_) also the temporal jitter (*s*_*i*_) of the synchronous pulse in the *i*th layer and investigate the two-dimensional iterated map for (*g*_*i*_, *s*_*i*_) (e.g., Diesmann et al., 1999; Gewaltig et al., 2001; Goedeke and Diesmann, 2008). However, even if the synchronous pulse is blurred out to a pulse packet with finite width, for sufficiently large connectivity stable propagation still can be obtained (see e.g., Gewaltig et al., 2001).

**Figure 11:**
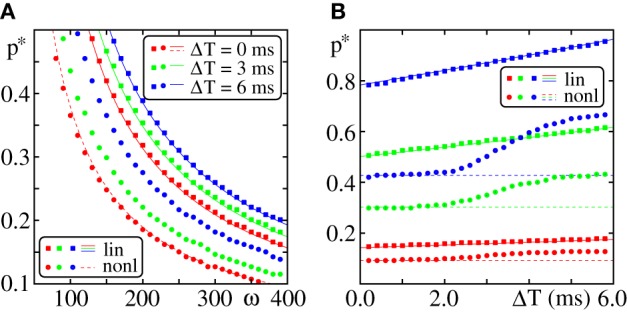
**Robustness against heterogeneities in the response delays**. **(A)** Critical connectivity vs. layer size for FFNs (*m* = 20, ϵ = 0.25 mV, *I*_0_ = 5 mV, ϵ^ext^ = 0.5 mV) with additive (squares) and non-additive (Θ_*b*_ = 4 mV, κ = 11 mV, Δ*t* = 2.5 ms; circles) dendritic interactions. Different colors indicate different widths of the delay distribution (cf. Equation 84). The solid lines indicate the critical connectivity p^*^_L_ corrected for inhomogeneous delay distribution (cf. Equation 90), the dashed line p^*^_NL_ for Δ*T* = 0 ms. **(B)** Critical connectivity vs. width of delay distribution Δ*T*. Different colors indicate different setups of the FFN (red: ω = 275, ϵ = 0.4 mV; green: ω = 125, ϵ = 0.25 mV; blue: ω = 200, ϵ = 0.1 mV). Solid and dashed lines are p^*^_L_ and p^*^_NL_ as before.

For linearly coupled FFNs, with increasing width of the delay distribution, Δ*T*, the propagating pulse becomes broader and thus the critical connectivity p^*^_L_ increases (cf. Figures 11A,B; squares). However, the scaling with layer size (cf. Figure 11A) and coupling strength (data not shown) is the same.

Under the assumption that the width of the pulse packet stays bounded, one can derive a lower bound for the critical connectivity. We assume that a pulse in layer *i* is perfectly synchronized and calculate the effective peak of the depolarization in the (*i* + 1)th layer. Replacing the coupling strength ϵ by the effective depolarization ϵ′ (derived below, cf. Equation 89) in the estimate of the critical connectivity (cf. Equation 32) one gains an estimate of the critical connectivity for systems with heterogeneous delays [Equation (90); shown in Figure 11]. Consider a perfectly synchronized pulse in layer *i*. Due to inhomogeneities in the delay, the inputs arriving at the (*i* + 1)th layer are distributed uniformly in an interval of size Δ*T* (Equation 83). We assume that all inputs arriving at a neuron of layer *i* + 1 are equidistantly distributed over [−Δ*T*/2, Δ*T*/2], i.e., the arrival time of the *l*th of a total number of *k* inputs is

(84) $$t_{l}^{\text{arr}}=\tau -\frac{\Delta T}{2}+\frac{\Delta T}{k-1}\cdot \left(l-1\right).$$

We consider the subthreshold dynamics only. Each single input depolarizes the neuron by an amount ϵ and afterwards the membrane potential *V*(*t*) decays exponentially toward its asymptotic value (*I*_0_) with the membrane time constant τ^m^ (cf. Equations 1, 2) until the next input arrives after a time interval $\frac{\Delta T}{k-1}$ (cf. Equation 84). Thus the total (effective) depolarization caused by the sum of these *k* inputs at the end of the considered time interval $\left(\tau +\frac{\Delta T}{2}\right)$ is

(85) $$\Delta \epsilon_{k}=\sum_{l=1}^{k}\epsilon \exp\left(-\frac{1}{\tau^{\text{m}}}\frac{\Delta T}{k-1}\left(l-1\right)\right)$$

(86) $$=\epsilon \frac{\exp\left(-\frac{\Delta T}{\tau^{\text{m}}}\frac{k}{k-1}\right)-1}{\exp\left(-\frac{\Delta T}{\tau^{\text{m}}}\frac{1}{k-1}\right)-1}.$$

We consider the effective depolarization per input, ϵ′, in the limit of a large number of inputs *k* (*k* → ∞),

(87) $$\epsilon^{\prime }=\underset{k\to \infty }{\lim}\left(\frac{\Delta \epsilon_{k}}{k}\right)$$

(88) $$=\frac{\tau^{\text{m}}}{\Delta T}\left(1-\exp\left[-\frac{\Delta T}{\tau^{\text{m}}}\right]\right)\epsilon$$

(89) =:C(ΔT)ϵ.

Thus the correction factor *C*(Δ*T*) ≤ 1 defined in Equation (90) relates the coupling strength ϵ to the effective coupling strength ϵ′ in the presence of inhomogeneous delays. The critical connectivity is then given by (cf. Equation 32)

(90) $$p_{L}^{*}=\frac{1}{C\left(\Delta T\right)}\cdot \frac{1}{\lambda^{*}\epsilon \omega }$$

and this estimate agrees well with direct numerical simulations (cf. Figure 11).

For FFNs with dendritic non-linearities and inhomogeneous delays τ_*kl*_, one has to consider a finite dendritic integration window Δ*t*^*d*^. Instead of amplifying only simultaneously received spikes (cf. Equation 5), the sum of spikes within the time interval Δ*t* is considered. We denote the sum of inputs to a neuron within the time interval [*t* − Δ*t, t*] by

(91) $$S_{k}^{\Delta t}(t)=\sum_{l}\sum_{m}\epsilon \chi_{\left[t-\Delta t,t\right]}\left(t_{lm}^{f}+\tau_{kl}\right),$$

where

(92) $$\chi_{A}(x)=\{\begin{array}{ll} 1 & \text{if }x\in A\\ 0 & \text{if }x\notin A \end{array}$$

is the indicator function and *t*^*f*^_*lm*_ is the *m*th firing time of neuron *l* as before. If *S*^Δ*t*^_*k*_(*t*) exceeds the dendritic threshold Θ_*b*_ for some *t* = *t*_0_, neuron *k* is depolarized additionally (to the depolarization arising from linear spike summation) by

(93) $$\epsilon_{\kappa }^{\text{add}}\left(t_{0}\right)=\kappa -S_{k}^{\Delta t}\left(t_{0}\right)$$

such that the total (effective) depolarization caused by an input *x* ≥ Θ_*b*_ equals κ, modeling the effect of a dendritic spike; cf. also section 3.3.3. After such an additional depolarization the dendrite becomes refractory for a time *t*^ref,ds^ and does not transfer additional spikes within the interval [*t*_0_, *t*_0_ + *t*^ref,ds^]. For Δ*t* = 0 we recover the non-linear modulation function σ_*NL*_(·) given by Equation (4). Due to the finite dendritic interaction window, a delay distribution with Δ*T* ≤ Δ*t* affects the critical connectivity only weakly (cf. Figure 11B). For Δ*T* > Δ*t*, some of the inputs received from the preceding layer upon a propagation of synchrony fall out of the dendritic interaction window Δ*T* and thus the critical connectivity increases. However, the scaling with layer size ω (cf. Figure 11B) and coupling strength ϵ (data not shown) is practically identical with the scenario Δ*T* = 0.

Before we discuss propagation of synchrony in biologically more plausible neuron models in section 3.3.3, we consider generalization of the non-linear modulation function in the following section.

#### 3.3.2. Coexistence of linear and non-linear propagation

**Figure 12:**
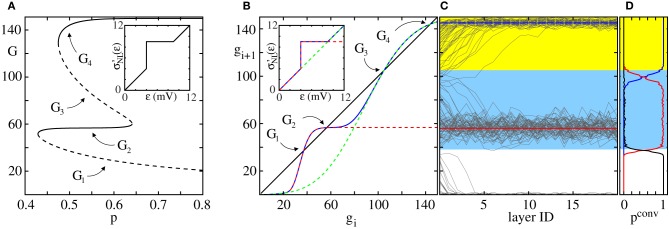
**Coexistence of linear and non-linear propagation**. **(A)** Bifurcation diagram obtained from Equation (13) for an FFN (ω = 150, ϵ = 0.225 mV) with a non-linear modulation function σ′_*NL*_ with incomplete saturation [cf. Equation (94) and inset]. Panel **(B)** shows the iterated maps (Equation 13) for *p* = 0.5 with the different non-linear modulation functions considered in this article (linear coupling: green,dashed; non-linear coupling σ_*NL*_: red, dashed; modified non-linear coupling σ′_*NL*_: blue). Panel **(C)** depicts the development of the size of the synchronous pulse along the layers of the FFN (single trials). The blue and yellow regions are the basins of attraction of *G*_2_ and *G*_4_, respectively, derived from the data in panel **(B)**. Panel **(D)** shows the probability *p*^conv^ of converging to the linear propagation regime (yellow area, blue line) and the non-linear propagation regime (blue area, red line) after *m* = 20 layers (*p*^conv^ is obtained from *n* = 150 runs with different networks and initial conditions).

In this article, we employed a non-linear modulation function σ_*NL*_(ϵ) that is linear for dendritic stimulation smaller than the dendritic threshold, ϵ < Θ_*b*_, and constant (i.e., saturates) for supra-threshold stimulation, ϵ ≥ Θ_*b*_ (cf. Equation 4). Biologically, if the linear inputs are transmitted despite the dendritic sodium spike and are not shadowed by, e.g., an NMDA spike, they may lead to a second, later peak depolarization after the one generated by the sodium spike. Since our models replace depolarizations by jumps to the peak depolarization, we have to account for the later peak as soon as it exceeds the earlier one. In this part, we thus assume that if the synchronous input is so large that the depolarization it generates upon linear summation exceeds the depolarization κ generated by the dendritic spike, this former is considered as the effect of the input. In other words, we assume that the dendritic modulation function continues linearly beyond κ, i.e., we define

(94) $$\sigma_{NL}^{\prime }(\epsilon )=\{\begin{array}{lll} \epsilon & \text{for} & \epsilon \leq \Theta_{b}\\ \kappa & \text{for} & \Theta_{b}\leq \epsilon \leq \kappa \\ \epsilon & \text{for} & \epsilon \geq \kappa \end{array}$$

(cf. inset of Figure 12A).

The iterated map, mapping the number of active neurons in layer *i* to the average number of active neurons in layer *i* + 1 may now have (depending on the system parameters) between one and five fixed points (cf. Figure 12). As before, *G*_0_ = 0 is a trivial fixed point corresponding to the level of absent activity and the only fixed point of the iterated map for small connectivity *p*. With increasing connectivity *p*, two additional pairs of fixed points *G*_1_ ≤ *G*_2_ and *G*_3_ ≤ *G*_4_ appear via tangent bifurcations. The first pair of fixed points, *G*_1_ and *G*_2_, correspond to the propagation of synchrony mediated by non-additive dendritic interactions (as discussed in section 3.1), the second pair, *G*_3_ and *G*_4_, correspond to propagation of synchrony mediated by linearly processed inputs (as discussed in section 3.2). By further increasing the connectivity *p*, the fixed points *G*_2_ and *G*_3_ disappear via a tangent bifurcation (cf. Figure 12A). Within the region, where five fixed points exists, both types of propagation of synchrony coexists (illustrated in Figures 12B–D): Synchronized pulses of size *g*_0_ < *G*_1_ typically decay to zero after a small number of layers. Pulse sizes with *G*_1_ < *g*_0_ < *G*_3_ typically initiate propagation of synchrony with an average pulse size around *G*_2_ (where the propagation is mediated by non-additive dendritic interactions) and synchronous pulses of size *g*_0_ > *G*_3_ typically initiate propagation of synchrony with average pulse sizes around *G*_4_ (linear propagation). For sufficiently large *p*, i.e., the fixed points *G*_2_ and *G*_3_ disappeared, a synchronized pulse of size *g*_0_ ≥ *G*_1_ will initiate propagation of synchrony with pulse sizes around *G*_4_; in this parameter region the non-additive dendritic interactions essentially increase the basin of attraction of *G*_4_.

Within the framework of our analytical tractable model, we neglect, e.g., the initiation time of a dendritic spike (in our model non-linear amplifications are instantaneous) or the different shapes of potential deflections caused by linearly and non-linearly processed inputs. Therefore, propagating synchronous signals mediated either by linear or non-linear dendrites differ only in their size. In biological more detailed models (briefly discussed in section 3.3.3 below) both propagation types will be more distinct, e.g., the propagation frequency (speed) and the quality of synchrony of the propagating pulses are different (see also Jahnke et al., 2012).

#### 3.3.3. Biological more detailed models

The model we mainly consider in this article has the advantage of being analytically tractable. Here we ask whether it over-simplifies the considered systems. More precisely, we study whether the results derived above, in particular the analytical estimates for the critical connectivity, generalize to biologically more detailed models.

The main assumption underlying our analysis of linearly coupled networks is a very general one, namely that synchronous single inputs sum up linearly: we assumed that the spiking probability *p*_*f*_(·) of a neuron due to the reception of *x* synchronous inputs of size ϵ equals the spiking probability due to the reception of one single input of size *y* = *x*ϵ. Therefore, the results will hold also for more complex neuron models, as long as the effect of a synchronous input pulse is approximately the sum of the effects of single inputs. In particular, if the spiking probability due to an input of strength *x, p*_*f*_(*x*), is sufficiently slowly changing with *x*, according to Equation (24) the critical connectivity scales like p^*^_L_ ∝ (ϵω)^−1^ for sufficiently large layer sizes and small coupling strengths. To fully compute the critical connectivity, the actual form of *p*_*f*_(·) has to be known. Our leaky integrate-and-fire neuron with infinitesimally short current pulses approximates the behavior of a wide class of neuron models for which an analytical derivation of *p*_*f*_(·) is impossible. Still even for more detailed models, *p*_*f*_(·) is accessible for measurements in single neuron (computer) experiments.

**Figure 13:**
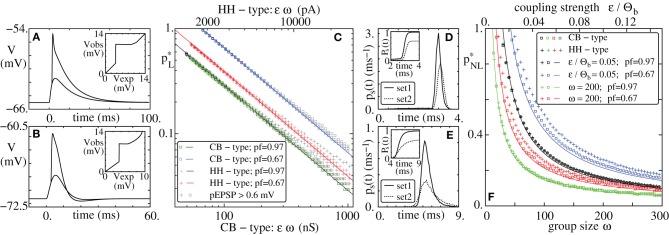
**Same scaling of propagating regime for networks of biologically more detailed neuron models**. **(A,B)** Time course of the membrane potential of single neurons receiving inputs that are sufficiently strong to elicit a dendritic spike, with (non-linear model) and without (linear model) dendritic spike generation mechanism, for **(A)** a conductance based LIF-type neuron (henceforth: CB-type), and **(B)** a Hodgkin–Huxley-type neuron (HH-type). The insets show the observed peak of the induced postsynaptic potential (pEPSP) vs. the pEPSP expected from linear input summation (equivalent to the dendritic modulation function in the analytically tractable model). **(C)** Critical connectivity p^*^_L_ vs. ϵω in linearly coupled networks. For each value ϵω, we evaluated the critical connectivity for four different group sizes ω = 100, 300, 500, 700 and four different coupling strengths ϵ = 0.3, 0.6, 0.9, 1.2 nS (CB-type; squares; lower horizontal axis) and ϵ = 9, 18, 27, 36 pA (HH-type; crosses; upper horizontal axis), respectively. The lines are fitted functions of the form (λϵω)^−1^. The analytical estimate given by Equation (24) holds in the limit of large layer sizes ω and small couplings ϵ, therefore we exclude data points from the fitting where a single input yields an EPSP larger than 0.6 mV (CB-type: ϵ ≥ 1.4 nS; HH-type: ϵ ≥ 46 pA; these points are marked in gray). **(D,E)** Probability distribution of somatic spike times after stimulation of the neuron by an input which is sufficiently strong to generate a dendritic spike (**D**: CB-type, **E**: HH-type). We show exemplary two different configurations for the external inputs, which result in a total somatic spiking probability after dendritic spike generation of *p*^*f*^ ≈ 0.97 (solid lines; set 1) and *p*^*f*^ ≈ 0.67 (dashed lines; set 2). *p*^*f*^ equals the saturation level of the corresponding cumulative distribution function (shown in the insets). **(F)** Critical connectivity p^*^_NL_ vs. group size ω (lower horizontal scale) and coupling strength ϵ normalized by threshold Θ_*b*_ (upper horizontal scale), respectively. The theoretical estimate of p^*^_NL_ (cf. Equation 71) is a function of ω, Θ_*b*_/ϵ and *p*^*f*^, therefore the predictions agree for both models and the data from direct numerical simulations are consistent with the theoretical predictions. [All simulations of FFNs in this figure are obtained for inhomogeneous delay distribution with Δ*T* = 1 ms (cf. Equation 83)].

In Figure 13 we verify our predictions exemplary for two types of neuron models: We employ a model of conductance based leaky integrate-and-fire-type neurons with exponential input conductances (CB-type; see Appendix) and a Hodgkin-Huxley-type neuron model with alpha-function shaped input currents (HH-type; see Appendix). The post-synaptic potential induced by single excitatory inputs is shown in panels (a) and (b) and the scaling of the critical connectivity p^*^_L_ with ϵω in panel (c): the scaling of p^*^_L_ is well described by p^*^_L_ ∝ (ϵω)^−1^.

The main assumptions underlying our analysis of non-linearly coupled networks are (1) that the maximal spiking probability due to inputs which are subthreshold relative to the dendritic threshold, *p*_*f*_ (Θ_*b*_), is significantly smaller than the spiking probability due to a suprathreshold input, *p*_*f*_ (κ), and (2) that the temporal jitter of somatic spikes evoked by suprathreshold inputs is small such that synchronized inputs stay synchronized. Both conditions have been found to be satisfied in biological neurons (e.g., Ariav et al., 2003). Therefore, Equation (71) specifying the critical connectivity p^*^_NL_ also holds for more detailed neuron models if these models incorporate biologically plausible features of fast dendritic spikes. To obtain a quantitative prediction of p^*^_NL_, it is sufficient to estimate (a) the number of inputs needed to elicit a dendritic spike, Θ_*b*_/ϵ, (b) the layer size ω, and (c) the spiking probability due to the reception of a total input that is sufficiently strong to elicit a dendritic spike.

To investigate the scaling of the critical connectivity p^*^_NL_ in direct numerical simulations, we account for the effects of dendritic spikes in the CB-type and HH-type: When the total excitatory input within the dendritic integration window exceeds the dendritic threshold level, a current pulse modeling the effect of a dendritic spike is initiated and causes an additional depolarization of the soma of the post-synaptic neuron (see Appendix for details; cf. also section 3.3.1). In Figure 13 we compare the results of direct numerical simulations with the estimate given by Equation (72). The post-synaptic potential induced by single excitatory inputs is shown in panels (A) and (B). Panel (D) and (E) shows the spiking probability of a single neuron (in the ground state of the FFN), *p*^*f*^, due to an input exceeding the dendritic threshold level; as examples we present two different setups with *p*^*f*^ = {0.67, 0.97}. Panel (F) shows the scaling of p^*^_NL_ with layer size and coupling strength and the good agreement of the analytical estimate with direct numerical simulations.

## 4. Summary and conclusions

Propagation of synchrony in feed-forward sub-structures that are embedded in randomly connected recurrent networks has been a research topic for more than two decades now [see, e.g., review on this topic (Kumar et al., 2010)] and it is hypothesized that such propagation possibly explain the emergence of spatio-temporal spike patterns and information transmission.

In this article, we have analyzed diluted FFNs and investigated their capability to propagate synchrony. In addition to conventional additive (linear) input processing at single neurons, we considered non-additive dendritic interactions modeling the impact of fast dendritic spikes (Ariav et al., 2003; Gasparini et al., 2004; Polsky et al., 2004; Gasparini and Magee, 2006). We emulated the influence of the embedding recurrent network which establishes the irregular ground state in the FFN, by random Poissonian inputs (van Vreeswijk and Sompolinsky, 1996, 1998; Brunel, 2000). This approach does not account for back-reactions of activity within the FFN on the embedding network. It is justified as long as the connectivity and connection strength between the neurons of the FFN and the embedding network is low and weak compared to the feed-forward connectivity and connection strength. The back-reaction then influences the activity of the embedding network only weakly and a robust propagation of synchrony can be achieved (Vogels and Abbott, 2005; Kumar et al., 2008; Jahnke et al., 2012). Yet, if the condition is not met, synchronous activity within the FFN may spread out over the embedding network and potentially cause pathological activity (“synfire-explosions”) (Mehring et al., 2003). For specifically structured networks also more complex interactions are possible, such as an enhancement of propagating synchrony (manuscript in preparation).

In the main part of the article, we studied the propagation of synchrony employing leaky integrate-and-fire neurons in the limit of temporally short synaptic inputs and homogeneous synaptic delays. Synchronous pulses consist of exactly synchronized (simultaneous) spikes. This allows to investigate propagation of synchrony by considering the size of a synchronized pulse only, so that the analysis becomes analytically tractable. Nevertheless, in the second part of our article we also consider systems with heterogeneous coupling delays and temporally extended interactions. In agreement with the literature (e.g., Diesmann et al., 1999; Gewaltig et al., 2001; Goedeke and Diesmann, 2008), we observe that pulse packets tend to synchronize along the layers of the FFN so that the results of our simplified description are directly applicable.

We derived scaling laws as well as quantitative estimates for the critical connectivity marking the bifurcation point between the regime where robust propagation of synchrony is possible and where it is not. In particular, based on a suitable series expansion we have shown that for linearly coupled FFNs the critical connectivity decays inversely proportional to layer size and coupling strength. Moreover, the proportionality factor can be estimated from the ground state properties of the single neurons. The estimate agrees with direct numerical simulations within the biologically relevant parameter regime where (a) the spontaneous firing rate of the neurons is low and (b) the distribution of membrane potentials is broad (each neuron receives a huge number of almost random presynaptic inputs). If a synchronous pulse propagates along the layers of a linearly coupled FFN, most of the neurons of each layer participate in the propagation of synchrony, independent of the actual layer size, coupling strength or layout of the external network.

For neurons incorporating non-additive dendritic interactions, the spiking probability as a function of the dendritic stimulation becomes discontinuous. Therefore, the analytical estimation of the critical connectivity in non-linearly coupled FFNs required a different approach than the treatment of linearly coupled FFNs. We have shown that the critical connectivity decays inversely proportional to the layer size (as in linearly coupled FFNs), and we have derived the dependence on the coupling strength which is more complicated. The critical connectivity is completely determined by layer size, spiking probability of the single neuron upon the reception of a non-linearly enhanced presynaptic input and the number of inputs required to reach the dendritic threshold. Our results indicate that in presence of non-linear dendrites, neurons process synchronous inputs similar to threshold units. Such units have been previously used as simplified rate neuron models to study activity propagation in discrete time, e.g., in Nowotny and Huerta (2003); Leibold and Kempter (2006); Cayco-Gajic and Shea-Brown (2013). Because the non-linear modulation function saturates, FFNs with non-additive dendritic interactions allow for a sparser coding, i.e., only a sub-fraction of each layer (the actual size depends on the non-linear enhancement level) participates in the propagation of synchrony. Whereas stable propagation of synchrony is possible in systems with and without dendritic non-linearities, it occurs in non-linearly coupled FFNs with substantially reduced feed-forward anatomy (reduced connectivity or reduced coupling strength) compared to linearly coupled FFNs.

The analytic derivation of the critical connectivity is based on rather general assumptions: (a) the effect of a synchronous input pulse is approximately the sum of the effects of single inputs and (b) for networks with non-additive dendritic interactions the spiking probability due to non-linearly enhanced input is substantially larger than due to a non-enhanced input. Therefore the predictions and estimates are directly applicable to networks of biologically more detailed neuron models.

In our article we have shown that even highly diluted feed-forward structures are suitable to reliably support the directed and constrained propagation of synchronous activity. Such structures occur naturally in sparse, random recurrent networks which are typical for the cortex. These structures might be enhanced by simple synaptic plasticity to enable synchrony propagation. Fast dendritic spikes promote this propagation, as they selectively amplify synchronous inputs and are only weakly influenced by irregular background activity.

Indeed, important candidate regions for the generation of propagating synchrony such as the hippocampus and other, neocortical regions exhibiting replay of activity (Nadasdy et al., 1999; Lee and Wilson, 2002; Ji and Wilson, 2007; Xu et al., 2011; Eagleman and Dragoi, 2012) are sparse and show synaptic plasticity (Debanne et al., 1998; Kobayashi and Poo, 2004). Dendritic spikes as prominently found in, e.g., the hippocampus (Ariav et al., 2003; Gasparini et al., 2004; Polsky et al., 2004; Gasparini and Magee, 2006) trigger depolarizations and calcium influx sufficient to change synaptic strengths (Golding et al., 2002; Remy and Spruston, 2007) and the dendrites itself exhibit branch “strength potentiation,” i.e., the strength of a dendritic spike on a dendritic branch exhibits experience- and activity-dependent plasticity (Losonczy et al., 2008; Makara et al., 2009; Müller et al., 2012).

Our work indicates that fast dendritic spikes reduce the required synaptic strength and connection density for replay of spike patterns. Moreover, their saturation and the resulting sparse coding might explain the observed variability during replay. Thus, in particular, our understanding of propagation along diluted feed-forward chains may now be combined with knowledge on synaptic plasticity and generation of activity accompanying replay (e.g., sharp wave/ripples) to gain an integrated mechanistic understanding for encoding, replay and memory transfer.

### Conflict of interest statement

The authors declare that the research was conducted in the absence of any commercial or financial relationships that could be construed as a potential conflict of interest.

## A. Appendix

### A.1 Proof of existence of a global minimum of *p*_*NL*_(*n*)

We will show that *p*_*NL*_(*n*) as derived in Equation (59),

(A.1) $$p_{NL}(n)=\frac{n^{2}\epsilon +2\Theta_{b}+n\sqrt{n^{2}\epsilon^{2}+4\Theta_{b}\left(\epsilon -\frac{\Theta_{b}}{\omega }\right)}}{p_{f}(\kappa )\epsilon (n^{2}+\omega )\left(1+\text{Erf}\left(\frac{n}{\sqrt{2}}\right)\right)}$$

(A.2) $$=\frac{1}{p_{f}(\kappa )}\frac{2\Theta_{b}+n^{2}\epsilon \left(1+\sqrt{1+\frac{\alpha }{n^{2}}}\right)}{\left(1+\text{Erf}\left(\frac{n}{\sqrt{2}}\right)\right)\left(n^{2}\epsilon +\omega \epsilon \right)},$$

has a global minimum for ϵω > Θ_*b*_. In Equation (A.2) we defined

(A.3) $$\alpha :=\frac{4\Theta_{b}}{\epsilon }\left(1-\frac{\Theta_{b}}{\epsilon \omega }\right).$$

For ϵω > Θ_*b*_, *p*_*NL*_ is positive and continuous, and approaches

(A.4) $$\underset{n\to -\infty }{\lim}\left(p_{NL}(n)\right)=\infty ,$$

(A.5) $$\underset{n\to \infty }{\lim}\left(p_{NL}(n)\right)=\frac{1}{p_{f}(\kappa )},$$

in the limit of large/small *n*. Further, the derivative of *p*_*NL*_ can be written as

(A.6) $$\frac{d}{dn}p_{NL}(n)=\left(2-h_{1}(n)\right)h_{2}(n),$$

where we defined the functions

(A.7) $$\begin{array}{l} h_{1}(n)=\frac{1}{\epsilon \omega }(\frac{\sqrt{\frac{2}{\pi }}e^{-\frac{n^{2}}{2}}\left(n^{2}+\omega \right)\left(\frac{2\Theta_{b}}{\sqrt{\alpha +n^{2}}+n}+n\epsilon \right)}{1+\text{Erf}\left(\frac{n}{\sqrt{2}}\right)}\\ \;+\frac{4\Theta_{b}n}{\sqrt{\alpha +n^{2}}+n}+\frac{\alpha \epsilon \left(n^{2}+\omega \right)}{\alpha +n\left(\sqrt{\alpha +n^{2}}+n\right)}), \end{array}$$

(A.8) $$h_{2}(n)=\frac{\omega \left(\sqrt{\alpha +n^{2}}+n\right)}{p_{f}\left(\kappa \right)\left(\text{Erf}\left(\frac{n}{\sqrt{2}}\right)+1\right){\left(n^{2}+\omega \right)}^{2}}.$$

For *n* > 0 and ϵω > Θ_*b*_,

(A.9) α>0,

(A.10) $$h_{1}(n)>0,$$

(A.11) $$h_{2}(n)>0,$$

and in the limit of large *n*,

(A.12) $$\underset{n\to \infty }{\lim}h_{1}(n)=\frac{1}{\epsilon \omega }\left(0+2\Theta_{b}+\frac{\alpha \epsilon }{2}\right)$$

(A.13) $$=2\frac{2\Theta_{b}\omega \epsilon -\Theta_{b}^{2}}{\omega^{2}\epsilon^{2}}$$

(A.14) $$\underset{n\to \infty }{\lim}h_{2}(n)=0.$$

For ϵω > Θ_*b*_, *h*_1_(*n*) is smaller than two for sufficiently large *n* (cf. Equation A.13) and thus the derivative of *p*_*NL*_(*n*) becomes positive (cf. Equation A.6). Consequently *p*_*NL*_ approaches 1/*p*_*f*_ (κ) from below for large *n* (cf. also Equation A.5). This proves the existence of a global minimum of *p*_*NL*_(*n*), because *p*_*NL*_ > 1/*p*_*f*_ (κ) for sufficiently small *n* (cf. Equation A.4).

### A.2 Biological more detailed neuron models

In section 3.3.3 we consider biologically more detailed neuron models. In this appendix we present descriptions of these models including the parameters used for the numerical simulations in Figure 13. These simulations were done using NEST (Gewaltig and Diesmann, 2007), a simulator for spiking neural network models (available at http://www.nest-initiative.org). We implemented new model classes within the NEST framework to handle conductance-based leaky integrate-and-fire neurons with double exponential input conductances as well as non-linear dendritic interactions (source code available from Sven Jahnke).

#### A.2.1 CB-type model

The CB-type model is a leaky integrate-and-fire neuron with conductance based synapses, augmented with a mechanism for the generation of current pulses mimicking the effect of a dendritic spike (see also Memmesheimer, 2010; Jahnke et al., 2012). The subthreshold dynamics of the membrane potential *V*_*l*_ of neuron *l* obeys the differential equation

(A.15) $$\begin{array}{l} C_{l}^{\text{m}}\frac{dV_{l}(t)}{dt}=g_{l}^{L}\left(V_{l}^{\text{rest}}-V_{l}(t)\right)+g_{l}^{A}(t)\left(E^{\text{Ex}}-V_{l}(t)\right)\\ \;+g_{l}^{G}(t)\left(E^{\text{In}}-V_{l}(t)\right)+I_{l}^{\text{DS}}(t)+I_{l}^{0}. \end{array}$$

Here, *C*^*m*^_*l*_ is the membrane capacity, *g*^*L*^_*l*_ is the resting conductance, *V*^rest^_*l*_ is the resting membrane potential, *E*^Ex^ and *E*^In^ are the reversal potentials, and *g*^*A*^_*l*_(*t*) and *g*^*G*^_*l*_(*t*) are the conductances of excitatory and inhibitory synaptic populations, respectively. *I*^DS^_*l*_(*t*) models the current pulses caused by dendritic spikes and *I*^0^_*l*_ is a constant current gathering slow external and internal currents. The time course of single synaptic conductances contributing to *g*^*A*^_*l*_(*t*) and *g*^*G*^_*l*_(*t*) is given by the difference between two exponential functions (e.g., Dayan and Abbott, 2001) with time constants τ^*A*, 1^ and τ^*A*, 2^ for the excitatory and τ^*G*, 1^ and τ^*G*, 2^ for the inhibitory conductances. Whenever the membrane potential reaches the spike threshold Θ_*l*_, the neuron sends a spike to its postsynaptic neurons, is reset to *V*^reset^_*l*_ and becomes refractory for a period *t*^ref^_*l*_. Additionally to inputs from the preceding layer each neuron receives excitatory and inhibitory Poissonian input spike trains with rates ν^ex^ and ν^in^; single inputs have coupling strength ϵ^ex^ and ϵ^in^, respectively.

To account for dendritic spike generation, we consider the sum *g*_*l*, Δ*t*_ of excitatory input strengths (characterized by the coupling strengths), arriving at an excitatory neuron *l* within the time window Δ*t* for non-linear dendritic interactions,

(A.16) $$g_{l,\Delta t}(t)=\sum_{j}\sum_{k}\epsilon_{lj}\chi_{\left[t-\Delta t,t\right]}(t_{jk}^{f}+\tau ),\;$$

where χ_[*t* − Δ*t, t*]_ is the characteristic function of the interval [*t* − Δ*t, t*], *t*^*f*^_*jk*_ is the *k*th firing time of neuron *j* and τ denotes the synaptic delay. We denote the peak conductance (coupling strength) for a connection from neuron *j* to neuron *l* by *g*^max^_*lj*_. If *g*_*l*, Δ*t*_ exceeds a threshold *g*_Θ_, a dendritic spike is initiated and the dendrite becomes refractory for a time window *t*^DS,ref^. The effect of the dendritic spike is incorporated into the model by the current pulse that reaches the soma a time τ^DS^ thereafter. This current pulse is modeled as the sum of three exponential functions,

(A.17) $$I_{l}^{\text{DS}}(t)=c(g_{\Delta t})\left[-Ae^{-\frac{t}{\tau^{\text{DS},1}}}+Be^{-\frac{t}{\tau^{\text{DS},2}}}-Ce^{-\frac{t}{\tau^{\text{DS},3}}}\right],$$

with prefactors *A* > 0, *B* > 0, *C* > 0, decay time constants τ^DS,1^, τ^DS,2^, τ^DS,3^ and a dimensionless correction factor *c*(*g*_Δ*t*_), where *g*_Δ*t*_ is the summed excitatory input at the initiation time of the dendritic spike as given by Equation (A.16). The factor *c*(*g*_Δ*t*_) modulates the pulse strength, ensuring that the peak of the excitatory postsynaptic potential (pEPSP) reaches the experimentally observed region of saturation. At very high excitatory inputs, the conventionally generated depolarization exceeds the level of saturation, *c*(*g*_Δ*t*_) is zero and the pEPSP increases (cf. inset of Figure 13A).

#### Parameters for Figure 13

The single neuron parameters for the numerical simulations are *C*^m^_*l*_ = *C*^m^ = 400 pF, *g*^*L*^_*l*_ = *g*^*L*^ = 25 nS, *V*^rest^_*l*_ = *V*^rest^ = −65 mV, Θ_*l*_ = Θ = −50 mV, *t*^ref^_*l*_ = *t*^ref^ = 3 ms and *V*^reset^_*l*_ = *V*^reset^ = −65 mV. The reversal potentials are *E*^Ex^ = 0 mV and *E*^In^ = −75 mV and the time constants for the excitatory and inhibitory conductances are τ^*A*,1^ = τ^*G*,1^ = 2.5 ms and τ^*A*,2^ = τ^*G*,2^ = 0.5 ms. The parameters of the dendritic spike current are Δ*t* = 2 ms, *g*^Θ^ = 8.65 nS, τ^DS^ = 2.7 ms, *A* = 55 nA, *B* = 64 nA, *C* = 9 nA, τ^DS,1^ = 0.2 ms, τ^DS,2^ = 0.3 ms, τ^DS,3^ = 0.7 ms and *t*^ref, DS^ = 5.2 ms and the dimensionless correction factor is given by *c*(*g*) = max {1.5 − *g* · 0.053nS^−1^, 0}. For the first setup (*p*^*f*^ ≈ 0.97) we set *I*^0^_*l*_ = *I*^0^ = 250 pA, ν^ex^ = 2.4 kHz, ν^in^ = 0.6 kHz, ϵ^ex^ = 0.6 nS and ϵ^in^ = 6.6 nS; for the second setup (*p*^*f*^ ≈ 0.67) we set *I*^0^_*l*_ = *I*^0^ = 0 pA, ν^ex^ = 20 kHz, ν^in^ = 5 kHz, ϵ^ex^ = 0.6 nS and ϵ^in^ = −6.6 nS.

#### A.2.2 HH-type model

We employ a standard model provided by NEST (“hh_psc_alpha”; Hodgkin–Huxley type neuron with alpha-function shaped postsynaptic currents) and incorporated a dendritic spike current as in the CB-Model. The membrane potential *V*_*l*_ of neuron *l* obeys the differential equation

(A.18) $$\begin{array}{l} C_{l}^{\text{m}}\frac{dV_{l}(t)}{dt}=I_{l}^{\text{Na}}(t)+I_{l}^{\text{K}}(t)+I_{l}^{\text{L}}(t)+I_{l}^{0}\\ \;+I_{l}^{\text{ex}}(t)+I_{l}^{\text{in}}(t)+I_{l}^{\text{DS}}(t). \end{array}$$

For clarity we drop the index *l* in the following; all quantities refer to some neuron *l*. In Equation (A.18),

(A.19) $$I^{\text{Na}}(t)=g^{\text{Na}}m{\left(t\right)}^{3}h(t)\left[E^{\text{Na}}-V(t)\right]$$

(A.20) $$I^{\text{K}}(t)=g^{\text{K}}n{\left(t\right)}^{4}\left[E^{\text{K}}-V(t)\right]$$

(A.21) $$I^{\text{L}}(t)=g^{\text{L}}\left[E^{\text{L}}-V(t)\right]$$

specify the Na^+^ current, the K^+^ current and leak current. The dynamics of the gating variables *m, n* and *h* are governed by

(A.22) $$\frac{dm(t)}{dt}=\alpha_{\text{m}}(t)\left[1-m(t)\right]-\beta_{\text{m}}(t)m(t)$$

(A.23) $$\frac{dh(t)}{dt}=\alpha_{\text{h}}(t)\left[1-h(t)\right]-\beta_{\text{h}}(t)h(t)$$

(A.24) $$\frac{dn(t)}{dt}=\alpha_{\text{n}}(t)\left[1-n(t)\right]-\beta_{\text{n}}(t)n(t),$$

where the voltage dependencies are given by

(A.25) $$\alpha_{n}(t)=\frac{0.01\left[\tilde{V}(t)+55\right]}{1-\exp\left[-\frac{\tilde{V}(t)+55}{10}\right]}$$

(A.26) $$\beta_{n}(t)=0.125\cdot \exp\left[-\frac{\tilde{V}(t)+65}{80}\right]$$

(A.27) $$\alpha_{m}(t)=\frac{0.1\left[\tilde{V}(t)+40\right]}{1-\exp\left[-\frac{V(t)+40}{10}\right]}$$

(A.28) $$\beta_{m}(t)=4\cdot \exp\left[-\frac{\tilde{V}(t)+65}{18}\right]$$

(A.29) $$\alpha_{h}(t)=0.07\cdot \exp\left[-\frac{\tilde{V}(t)+65}{20}\right]$$

(A.30) $$\beta_{h}(t)={\left(1+\exp\left[-\frac{\tilde{V}(t)+35}{10}\right]\right)}^{-1}.$$

In Equations (A.25–A.30) $\tilde{V}(t):=\frac{V(t)}{1\text{mV}}$ is the value of membrane potential normalized by 1 mV. Spikes are detected by a combined threshold-and-local-maximum search, if there is a local maximum above a certain threshold of the membrane potential, *U*_Θ_ = 0 mV, it is considered a spike (for more details see the NEST manual and the model implementation available at http://www.nest-initiative.org). After a synaptic delay time τ a spike initiates an alpha-function shaped current pulse at the post-synaptic neurons. The total excitatory and inhibitory input to neuron *l* is given by

(A.31) $$I^{\text{ex}}(t)=\sum_{k}\epsilon_{k}^{\text{ex}}\frac{e}{\tau^{\text{ex}}}\exp\left[-\frac{t}{\tau^{\text{ex}}}\right]\Theta \left[t-t_{k}^{\text{ex}}\right]$$

(A.32) $$I^{\text{in}}(t)=\sum_{k}\epsilon_{k}^{\text{in}}\frac{e}{\tau^{\text{in}}}\exp\left[-\frac{t}{\tau^{\text{in}}}\right]\Theta \left[t-t_{k}^{\text{in}}\right],$$

where ϵ^ex^_*k*_ > 0 (ϵ^in^_*k*_ < 0) is the strength of the *k*th arriving excitatory (inhibitory) spike at neuron *l, t*^ex^_*k*_ (*t*^in^_*k*_) denotes the reception time of that spike and *e* is the Euler constant [the currents *I*^ex^(*t*) and *I*^in^(*t*) are normalized such that an input of strength ϵ = 1 pA causes a peak current of 1 pA]. The time constants τ^ex^ and τ^in^ are the synaptic time constants. As before, we account for dendritic spike generation by considering the sum of excitatory input strengths received by neuron *l* within the time window Δ*t*,

(A.33) $$\epsilon_{\Delta t}(t)=\sum_{k}\epsilon_{k}^{\text{ex}}\chi_{\left[t-\Delta t,t\right]}(t_{k}^{f}+\tau ).$$

If this sum exceeds the dendritic threshold *I*^Θ^, a dendritic spike is initiated and we model its effect is by the current pulse

(A.34) $$I^{\text{DS}}(t)=c(\epsilon_{\Delta t})\left[-Ae^{-\frac{t}{\tau^{\text{DS},1}}}+Be^{-\frac{t}{\tau^{\text{DS},2}}}-Ce^{-\frac{t}{\tau^{\text{DS},3}}}\right],$$

starting after a delay time τ^DS^ after the initiation time of the dendritic spike. The correction factor *c*(ϵ_Δ*t*_) modulates the pulse strength such that the depolarization saturates for suprathreshold inputs until the effects of linearly summed input exceed the effects of the dendritic spike (cf. inset of Figure 13B).

#### A.2.3 Parameters for Figure 13

As before, we consider homogeneous neuronal properties. The single neuron parameters for the numerical simulations are *C*^m^ = 200 pF, *E*^K^ = −77 mV, *E*^L^ = −70 mV, *E*^Na^ = 50 mV, *g*^K^ = 3600 nS, *g*^L^ = 30 nS, *g*^Na^ = 12000 nS, τ^ex^ = 2 ms and τ^in^ = 2 ms. The parameters of the dendritic spike current are Δ*t* = 3.5 ms, *I*^Θ^ = 270 pA, τ^DS^ = 2.7 ms, *A* = 27.5 nA, *B* = 32 nA, *C* = 4.5 nA, τ^DS,1^ = 0.2 ms, τ^DS,2^ = 0.3 ms, τ^DS,3^ = 0.7 ms and *t*^ref,DS^ = 5.2 ms and the dimensionless correction factor is given by *c*(ϵ) = max {1.54 − ϵ · 0.002 pA^−1^, 0}. For the first setup (*p*^*f*^ ≈ 0.97) we set *I*^0^ = 500 pA, ν^ex^ = 3 kHz, ν^in^ = 3 kHz, ϵ^ex^ = 20 pA and ϵ^in^ = −20 pA; and for the second setup (*p*^*f*^ ≈ 0.67) we set *I*^0^ = 250 pA, ν^ex^ = 10 kHz, ν^in^ = 10 kHz, ϵ^ex^ = 20 pA and ϵ^in^ = −20 pA.
